# From nature-inspired electron acceptors to BioAIE materials with polarity- and polymorphism-dependence for anti-counterfeiting

**DOI:** 10.1039/d5sc09027j

**Published:** 2025-12-15

**Authors:** Dongmei Wang, Xiao Chen, Yuting Lin, Lulu Wu, Zhichen Zhao, Xu Xu, Shilong Yang, Jianyu Zhang, Wen-Jin Wang, Zheng Zhao, Shifa Wang, Ben Zhong Tang, Xu-Min Cai

**Affiliations:** a Jiangsu Co-Innovation Center of Efficient Processing and Utilization of Forest Resources, College of Chemical Engineering, Nanjing Forestry University Nanjing 210037 China xumin.cai@njfu.edu.cn wangshifa65@163.com; b Guangdong Basic Research Center of Excellence for Aggregate Science, School of Science and Engineering, The Chinese University of Hong Kong Shenzhen Guangdong 518172 China tangbenz@cuhk.edu.cn; c Advanced Analysis and Testing Center, Nanjing Forestry University Nanjing 210037 China; d State Key Laboratory of Biobased Transportation Fuel Technology, Department of Polymer Science and Engineering, Zhejiang University Hangzhou 3100587 China zhangjianyu@zju.edu.cn; e Guangdong Provincial Key Laboratory of Luminescence from Molecular Aggregates Guangzhou 510640 China

## Abstract

Organic optical functional materials show immense potential in smart materials, bioimaging, and theranostics. Despite their widespread utility, most photofunctional materials are principally derived from petrochemical sources, facing limitations in sustainability and monotonous skeletal structure. Nature-derived compounds offer unique molecular scaffolds that can inspire the design of innovative optical functional materials in addition to renewability and sustainability. Herein, we report the rational design of a novel biomass-based electron acceptor, dehydroabietic acid quinoxaline (DAQx), derived from renewable rosin. By coupling DAQx with triphenylamine, we constructed a series of electron donor–acceptor-type natural product-based aggregation-induced emission materials with tunable conjugation and charge transfer characteristics. These compounds exhibit dual-state responsive fluorescence, demonstrating both solvent-dependent emission in solution and polymorphism-dependent luminescence in solids. Remarkably, DAQx-BP displays distinct green and yellow fluorescence in different crystalline polymorphs, despite near-identical molecular packing with intermolecular interaction differences of <0.01 Å, which is a rare phenomenon highlighting extreme structure–property sensitivity. Leveraging these unique photophysical properties, DAQx-BP is applied in dual-modal smart anti-counterfeiting in both solution and aggregate states. This work not only provides a general strategy for designing sustainable, natural product-derived electron acceptors but also significantly expands the functional applications of natural resources in advanced optical materials.

## Abstract 2

BioAIE materials, derived from a novel natural electron acceptor with unique polarity- and polymorphism-dependent properties, enable smart anti-counterfeiting.

## Graphical abstract

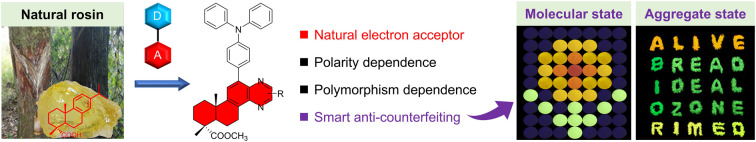

## Introduction

Organic functional materials have emerged as pivotal components in optoelectronic devices,^1,2^ biomedicine,^3,4^ and energy technologies,^5,6^ owing to their cost-effectiveness, structural tailorability, and tunable properties. Nature, as a master architect, has long inspired the design of high-performance functional materials—for instance, spider silk has inspired the development of bulletproof vests and surgical sutures, while chameleons and mimosa plants have stimulated the creation of stimuli-responsive smart materials. Among these, photofunctional materials occupy a particularly prominent position due to their unique optical properties, demonstrating transformative potential in advanced applications including sensors,^7–9^ bioimaging,^10–13^ theranostics,^14,15^ and intelligent anti-counterfeiting.^16,17^ However, most current photofunctional materials rely on petrochemical feedstocks, which suffer from sustainability challenges and limited structural diversity. Turning to nature's molecular repertoire could provide a compelling solution.

In alignment with global sustainability goals, biomass-based materials garnered significant scientific interest owing to their renewability, biocompatibility, and unique molecular scaffolds that make them particularly attractive for structural exploitation and functional development.^18–23^ Small-molecule natural products, such as quercetin, berberine, coumarin, and tanshinone IIA, have been extensively explored for their pharmaceutical and biological properties.^24–27^ Intriguingly, these compounds feature aromatic rings and flexible twisted structures, endowing them with aggregation-induced emission (AIE) properties.^28–30^ Likewise, natural polymers such as cellulose, hemicellulose, and lignin demonstrate room-temperature phosphorescence (RTP) due to their heteroatom-enriched compositions and rigid skeletal scaffolds.^31–33^ These naturally derived AIE (BioAIE)/RTP (BioRTP) materials exhibit remarkable potential across multiple advanced applications such as bioimaging,^30,34^ photodynamic therapeutics,^35,36^ and smart anti-counterfeiting.^31,37^ Collectively, these advances underscore nature's potential as a superior and sustainable source of molecular scaffolds for photofunctional materials, circumventing the limitations of conventional petrochemical-based systems.

Dehydroabietic acid is a tricyclic diterpenoid abundantly derived from disproportionated rosin, which is a major product of natural rosin. Its rigid alicyclic framework, when incorporated into Schiff base structures, effectively suppresses non-radiative decay pathways, enabling the construction of rosin-based AIE materials.^38,39^ Moreover, this tricyclic scaffold imparts stimuli-responsive behaviors such as mechanochromism, making it particularly attractive for dynamic anti-counterfeiting applications.^40,41^ However, current rosin-based BioAIE materials rely on simple Schiff base chromophores, which suffer from constrained luminescent performance, manifesting as limited emission diversity and weak fluorescence intensity. Electron donor–acceptor (D–A) engineering, a strategy for modulating the photophysical properties of luminescent materials,^42,43^ presents an effective approach to enhance the optoelectronic performance of rosin-based BioAIE materials. Notably, existing research on D–A type materials has predominantly focused on electron donor development, benefiting from their structural diversity and facile modifiability.^44,45^ In contrast, the number of strong electron-withdrawing acceptors remains relatively limited, presenting a key bottleneck in material design.^42^ Quinoxaline (benzopyrazine), a nitrogen-containing heterocyclic compound, not only serves as the core structure of many alkaloids (*e.g.*, triostin A)^46,47^ but also possesses a conjugated scaffold and moderate electron-withdrawing ability. Traditional studies on quinoxaline-based alkaloids have primarily focused on their medicinal value, while investigations into the optoelectronic properties derived from their inherently planar conjugated scaffolds remain scarce. By strategically integrating the structural merits of dehydroabietic acid (a rigid, biomass-derived scaffold) with quinoxaline's electron-deficient heterocycle, we envision a new class of sustainable luminescent materials with enhanced stimuli-responsive behavior. Such an € approach holds significant importance for advancing renewable and sustainable smart anti-counterfeiting materials with stimulus-responsive characteristics.

**Fig. 1:**
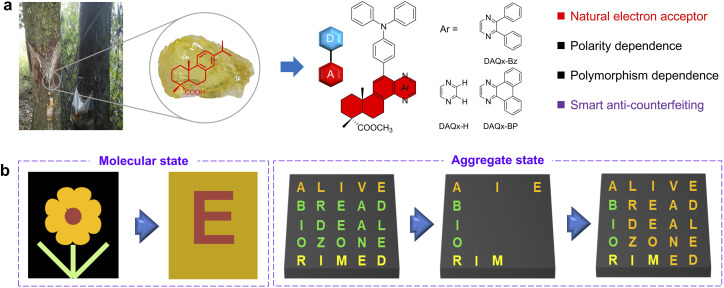
(a) Molecular design of rosin-derived D–A-type materials. (b) Dynamic encryption–decryption applications in molecular and aggregate states.

Herein, a novel natural electron acceptor, dehydroabietic acid-quinoxaline (DAQx), has been designed, deriving from the natural product dehydroabietic acid. Further, by using triphenylamine (TPA) as the electron donor, a series of D–A-type BioAIE compounds with different conjugation and intramolecular charge transfer (ICT) strengths based on DAQx have been constructed. These compounds exhibit both polarity- and polymorphism-dependent fluorescence, which clearly illustrates the influence of D–A-type structure on molecular and aggregate states. Interestingly, polymorphism samples of DAQx-BP have extremely similar conformations and packing with intermolecular interactions less than 0.01 Å, resulting in a significantly differentiated luminescence behavior of green and yellow fluorescence. Based on the unique polymorphism and acidichromism properties, DAQx-BP has been successfully applied to bimodal intelligent dynamic encryption–decryption and anti-counterfeiting. This work develops a novel natural electron acceptor, DAQx, and constructs polarity- and polymorphism-dependent BioAIE materials for smart anti-counterfeiting (Fig. 1).

## Results and discussion

### Molecular design, syntheses, and characterizations

**Scheme 1:**
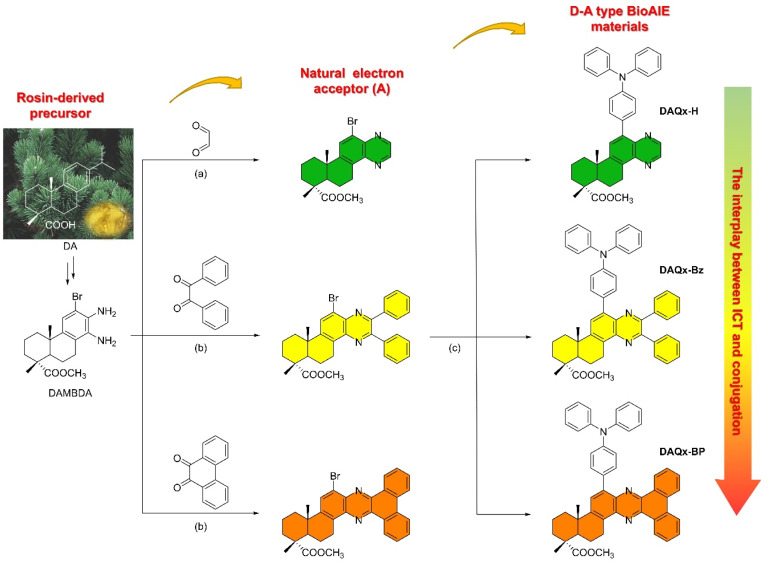
Synthesis routes of DAQx-H, DAQx-Bz, and DAQx-BP, respectively. (a) EtOH, reflux, 10 h. (b) CH_3_COOH, reflux, 12 h. (c) Pd(PPh_3_)_4_, K_2_CO_3_, toluene, N_2_, reflux, 5 h.

For D–A-type compounds, both the ICT effect and conjugation effect significantly influence their photophysical properties. Therefore, using the interplay between these two factors represents a robust strategy to design D–A type luminescent materials. To address this, a series of natural acceptors based on the dehydroabietic acid-quinoxaline skeleton (DAQx) are designed from dehydroabietic acid (DA), systematically varying ICT and conjugation effects to elucidate their relationship (Scheme 1). Furthermore, taking advantage of its propeller-shaped geometry and strong electron-donating capability, TPA is incorporated as both an electron donor and a rotor unit to construct D–A-type BioAIE materials. This design enables an in-depth investigation of the structure–property relationship between molecular aggregation and photophysical behavior. The synthesized compounds are thoroughly characterized by nuclear magnetic resonance (NMR) spectroscopy, high-resolution mass spectrometry (HRMS), and single-crystal X-ray diffraction (Fig. S1–S15 in the SI for details).

### Twisted intramolecular charge transfer and aggregation-induced emission properties

**Fig. 2:**
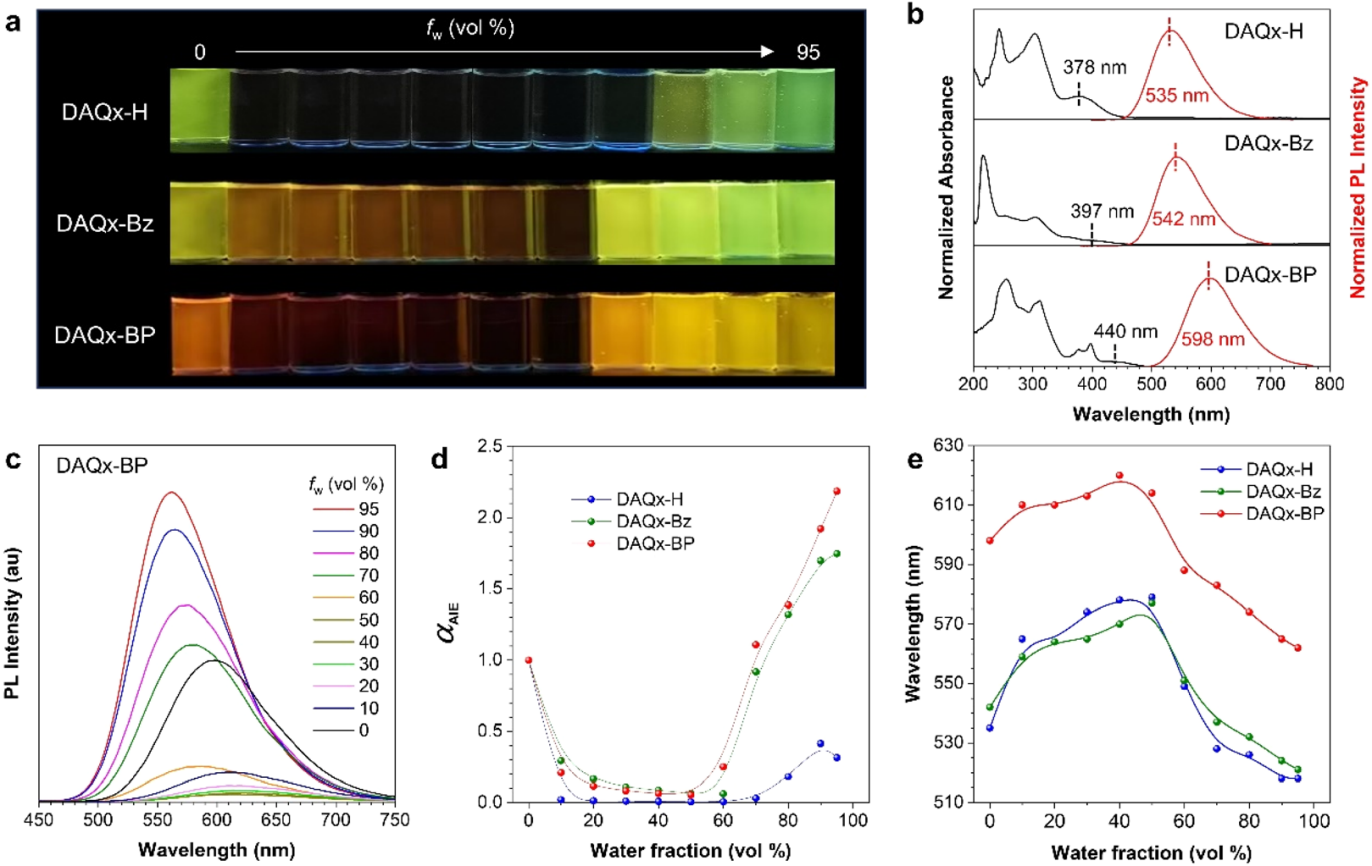
(a) Fluorescent photographs of DAQx-H, DAQx-Bz, and DAQx-BP in THF/H_2_O mixtures with different water fractions (*f*_w_) taken under 365 nm UV irradiation. (b) Normalized absorption and PL spectra of DAQx-H, DAQx-Bz, and DAQx-BP in pure THF solution. (c) PL spectra of DAQx-BP in THF/H_2_O mixtures with different *f*_w_. (d and e) The plots of the (d) *α*_AIE_ and (e) emission maximum *versus f*_w_ of DAQx-H, DAQx-Bz, and DAQx-BP. *α*_AIE_ = *I*/*I*_0_, *I*_0_ = PL intensity in pure THF. Concentration: 10 µM; Excitation wavelength: 378 nm for DAQx-H; 360 nm for DAQx-Bz, and 397 nm for DAQx-BP.

To investigate the photophysical properties of these compounds with the natural acceptor skeleton, their luminescent behavior in the tetrahydrofuran (THF)/H_2_O mixtures and organic solvents with different polarities is studied. First, they show distinct fluorescence changes in the THF/H_2_O mixed system with different water fractions (*f*_w_) intuitively (Fig. 2a). At the molecular state (*f*_w_ = 0%), all three compounds exhibit fluorescence with the maximum photoluminescence (PL) intensity at 535 nm, 542 nm, and 598 nm, respectively, accompanied by a redshift in absorption maxima from 378 nm (DAQx-H) to 397 nm (DAQx-Bz) and 440 nm (DAQx-BP) (Fig. 2b). These observations align with our molecular design strategy, confirming a gradual enhancement in D–A strength from DAQx-H to DAQx-BP. In addition, as the *f*_w_ increases from 10% to 60%, the fluorescence intensity sharply declines alongside a redshifted emission, attributable to increased solvent polarity, which can be classified as a twisted intramolecular charge transfer (TICT) state. In non-polar solvents (*f*_w_ = 0%), the planar molecular configuration, stabilized by electronic conjugation, emits fluorescence corresponding to the locally excited (LE) state. As solvent polarity increases, molecular conformation distorts, initiating charge separation and an excited-state transition from LE to TICT state, with concurrently decreased emission wavelength red-shift intensity. Further polarity elevation exacerbates donor–acceptor (D–A) moiety distortion, triggering complete intramolecular charge separation; polar solvents stabilize these charge-separated species. Owing to the elevated highest occupied molecular orbital (HOMO) energy levels and enhanced intramolecular motion, luminescence undergoes significant red-shifting and severe quenching. When *f*_w_ = 10%, the fluorescence of DAQx-H changes the most significantly, showing a trend of fluorescence quenching, indicating that it is most sensitive to polarity changes. As the *f*_w_ ≥ 70%, the fluorescence intensity of the three molecules shows an enhanced trend, and the wavelength undergoes a blueshift. It is attributed to the AIE characteristics after forming aggregates (confirmed by dynamic light scattering results, Fig. S16 and Table S1), which decreases the polarity inside the aggregates and suppresses intramolecular motions and non-radiative transitions (Fig. 2c–e and S17).^44,45,48,49^ Thus, the above results successfully prove these three compounds as D–A-type AIE luminogens with biomass-based electron acceptors.

### Synergistic effect of ICT and conjugation

**Fig. 3:**
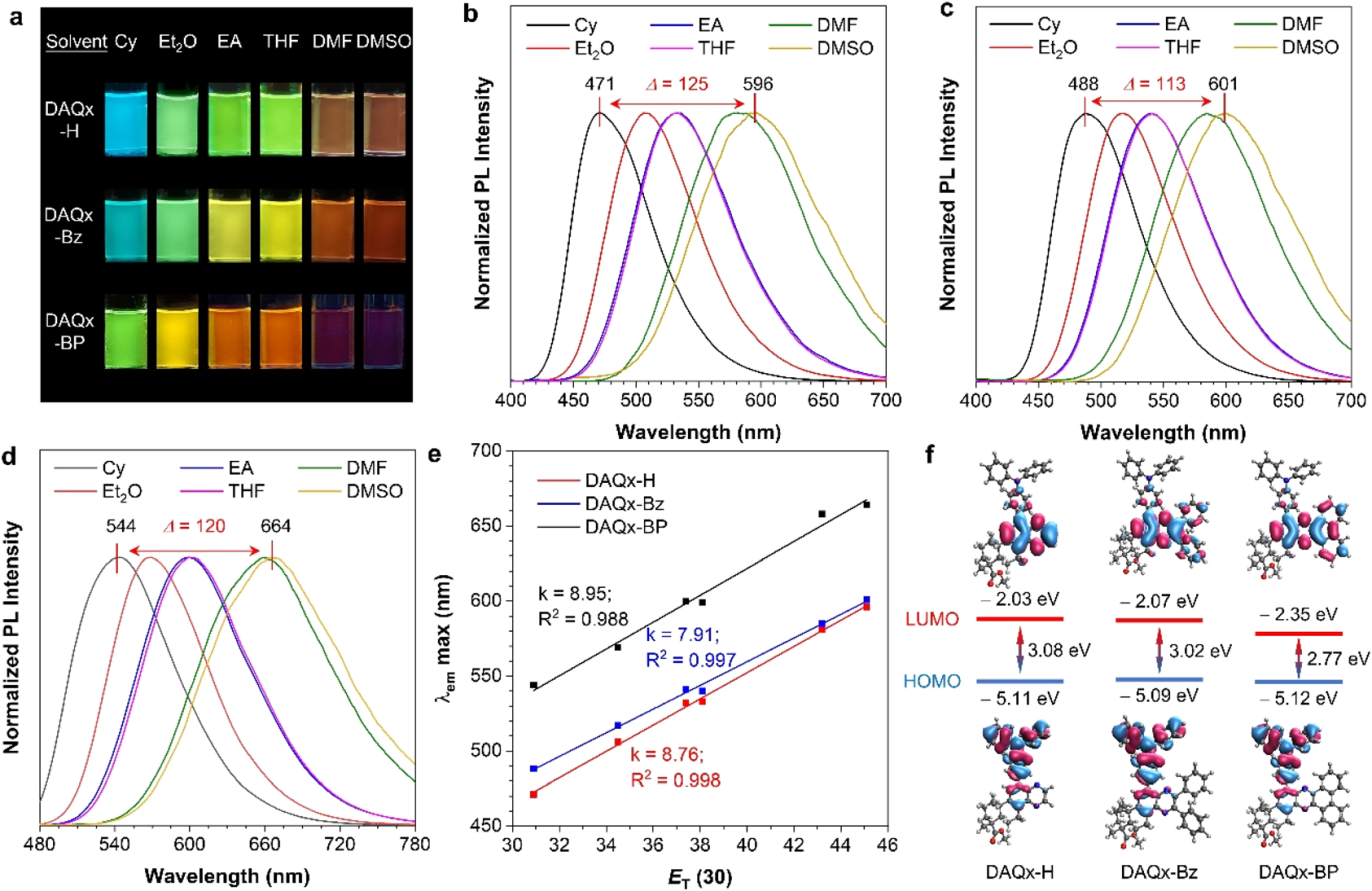
(a) Fluorescence photographs of DAQx-H, DAQx-Bz, and DAQx-BP in different solvents taken under 365 nm UV irradiation. (b–d) Normalized photoluminescence spectra of (b) DAQx-H, (c) DAQx-Bz, and (d) DAQx-BP in different solvents. Concentration: 10 µM. Δ represents the shift of emission wavelength of these compounds dissolved in DMSO and Cy. (e) The linear relationship between the maximum emission wavelength and the polarity parameter (*E*_T_(30)) of DAQx-H, DAQx-Bz, and DAQx-BP, respectively. (f) Frontier molecular orbitals and energy levels of DAQx-H, DAQx-Bz, and DAQx-BP, respective.

To better compare their D–A strength and provide a clear structure–property relationship, solvent-dependent luminescence is further studied (Fig. 3 and S18). All three compounds exhibit significant polarity-responsive characteristics (Fig. 3a). From cyclohexane (Cy) to dimethyl sulfoxide (DMSO) with the increased solvent polarity, a progressive bathochromic shift in emission wavelength is observed, accompanied by a reduction in fluorescence intensity (Fig. 3b–d). Comparative analysis reveals that introducing -Bz and -BP units enhances conjugation relative to DAQx-H, leading to redshifted emission. Specifically, DAQx-Bz, featuring two isolated benzene rings linked by a rotatable single bond, exhibits only a modest redshift due to restricted conjugation. In contrast, DAQx-BP adopts a rigid, planar conjugated structure due to fused benzene rings, significantly enhancing conjugation and yielding the most pronounced bathochromic shift. As a result, the emission wavelength difference value (Δ) of DAQx-H in two different solvents (*e.g.*, Cy and DMSO) is the largest (125 nm), followed by DAQx-BP (120 nm) and DAQx-Bz with the smallest value (113 nm). Meanwhile, a strong linear correlation (*R*^2^ > 0.988) exists between emission maxima and the solvent polarity parameter (*E*_T_(30)), with large regression slopes (*k* ≥ 7.91) confirming robust intramolecular charge transfer (ICT) and high sensitivity to environmental polarity (Fig. 3e). This finding is further corroborated by the Lippert–Mataga analysis, where the derived slopes quantitatively reflect the compounds' polarity sensitivity and D–A interaction strength (Fig. S19).

Theoretical calculation based on density functional theory (DFT) also supports the above analysis. As shown in Fig. 3f, the HOMO is located on the electron donor TPA with nearly identical energy levels (−5.09 to −5.12 eV), while the lowest unoccupied molecular orbital (LUMO) is concentrated on quinoxaline and derivative parts, clearly indicating their D–A structure. Specifically, DAQx-Bz shows only a marginal decrease (−0.04 eV) relative to DAQx-H, attributable to the limited conjugation enhancement and weak electron-donating effect of its two isolated benzene rings. In contrast, DAQx-BP displays a significant LUMO energy reduction by 0.32 eV, which is associated with the stronger conjugation effect endowed by the planar structure of the -BP unit. This electronic structure evolution directly correlates with the observed bathochromic shift in emission from DAQx-H to DAQx-BP. The above analysis confirms the feasibility of constructing D–A type molecules using DAQx skeleton as a novel natural electron acceptor and proves that both the ICT and conjugation effects have important effects on the photophysical properties, which can synergistically regulate the photophysical properties of D–A-type compounds.

### Dynamic decryption in the molecular state

**Fig. 4:**
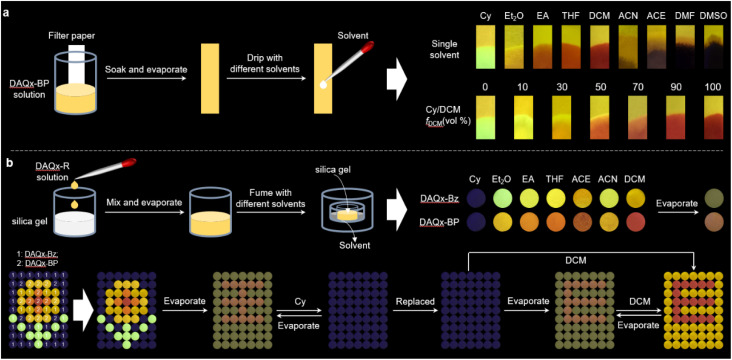
(a) Schematic diagram of fabricating solvent-test strips based on DAQx-BP and photographs of corresponding fluorescence changing from yellow to multicolor in response to different solvents. (b) Schematic diagram of fabricating silica gel mixed with DAQx-Bz and DAQx-BP as well as its potential applications for dynamic decryption.

Compounds exhibiting polarity-responsive luminescence demonstrate significant potential for environmental sensing and practical anti-counterfeiting applications. To exploit this property, a solvent-responsive test strip is successfully fabricated (Fig. 4a). The test strip is prepared by immersing a paper substrate in a DAQx-BP solution, followed by solvent evaporation, and the loading amount of DAQx-BP is calculated as 0.07 mg according to the standard curve of DAQx-BP in DCM (Fig. S20). Upon exposure to different solvents, the strip exhibits high-contrast fluorescence emission (*e.g.*, green, yellow, orange, red, and black), enabling rapid identification of solvent type and polarity. Furthermore, for binary solvent systems (*e.g.*, Cy/dichloromethane, DCM), the composition ratio can be semi-quantitatively estimated based on the fluorescence color shift—higher DCM fractions induce a pronounced redshift in emission. Similar to conventional pH-indicator strips, these solvent-test strips are facile to manufacture, highlighting the scalability of DAQx-based compounds for industrial applications. When immobilized on silica gel, DAQx derivatives display reversible multicolor fluorescence upon solvent vapor exposure (Fig. 4b and S21). The emission reverts to its initial state after solvent evaporation, confirming excellent cyclability. The time-dependent fluorescence and reversibility for silica gel doped with DAQx-Bz and DAQx-BP during fumigation with Cy and DCM solvents (Fig. S22–S25) are investigated. As shown in Fig. S22, when Cy is first used for fumigation, the fluorescence intensity of the sample gradually decreases over time until it is almost completely quenched. Subsequently, upon switching to DCM fumigation, the fluorescence intensity rapidly increases, accompanied by a significant red shift; the most noticeable changes occur within the first four minutes of fumigation. Continuing to observe the DCM evaporation stage, as the solvent gradually evaporates, the fluorescence intensity decreases synchronously, and the peak position shifts blue. This operation is repeated cyclically five times, and the results of each experiment are nearly identical, confirming that the stimulus response of DAQx derivatives on the silica gel substrate is reversible and cyclically stable (Fig. S25). These experimental results confirm that the sample exhibits good solvent-responsive efficiency and reversibility, indicating potential for application in solvent detection.

This stimulus-responsive behavior is further leveraged to achieve dynamic information encryption. Silica gel particles doped with DAQx-Bz and DAQx-BP are partitioned into discrete containers as fluorescent “pixels”. Firstly, silica gel doped with DAQx-Bz is fumigated with Cy, Et_2_O, and DCM, while silica gel doped with DAQx-BP is fumigated with Cy, Et_2_O, THF, and DCM. These treated silica gel samples are then arranged in a “flower” pattern. Once the solvents had completely evaporated, a Chinese character is identified. Subsequent Cy fuming erases all fluorescence, rendering the system informationally inert rearrangement of the containers yields no discernible pattern. However, after Cy evaporation, fluorescence recovers, revealing the encrypted letter “E.” Further, DCM fuming produces a yellow-emissive background with a red “E”, demonstrating multistage information encryption. These proof-of-concept demonstrations, solvent-responsive test strips, and dynamic decryption systems underscore the potential of rosin-derived DAQx compounds as sustainable, renewable materials for advanced anti-counterfeiting technologies.

In addition, we have also explored the responsiveness of DAQx derivatives to volatile organic compounds (VOCs) like chloroform (Chl), DCM, formaldehyde (HCHO), acetone (ACE), ethanol (EtOH), and toluene (Tol). As shown in Fig. S26, DAQx-BP loaded in filter paper shows an orange fluorescence darkened after ACE fumigation, shifts to yellow after EtOH fumigation, but remained largely unchanged with other solvents. DAQx-BP exhibits significant fluorescence responses under different solvent vapors when immobilized on silica gel: after Chl and DCM fumigation, the sample's fluorescence shifts from orange to red, with DCM inducing a more pronounced red shift; HCHO fumigation resultes in a yellow color, while EtOH fumigation produces bright yellow fluorescence. This study validates the stimulus-response performance of DAQx derivatives in saturated vapor environments of volatile organic compounds, as well as laboratory common organic reagents, and promises application potential to develop chemical sensors suitable for harmful environmental gases or industrial volatile solvent leaks.

Furthermore, we have expanded the experiment to evaluate the stimulus-response performance of the DAQx-BP on more diverse substrates, such as metallic substrates (like gold sheet), polymeric substrates (like PMMA film), and natural fiber substrates (like cotton threads). As shown in Fig. S27, the fluorescence characteristics of the DAQx-BP in metallic substrate remains almost unchanged before and after fumigation, with no significant differences observed, while exhibiting distinct changes in PMMA film after DCM and EA fumigation. Surprisingly, when cotton threads are used as substrates, DAQx-BP exhibited no significant fluorescence changes before or after solvent fuming. However, upon solvent addition, its fluorescence color shifted dramatically yet fully recovered to the original state after solvent evaporation. It indicates that natural fiber is also a suitable and convenient, and affordable substrate for DAQx derivatives to develop chemical sensors for industrial volatile solvent leaks.

### Photophysical properties in the solid state with unique polymorphism

The polarity-responsive luminescence of DAQx compounds in their molecular state presents significant potential for anti-counterfeiting applications. Based on their AIE characteristics illustrated in Fig. 2, the photophysical properties in the solid state are further investigated. DAQx-H and DAQx-Bz exhibit blue–green emission (500 nm), while DAQx-BP displays a red-shifted green emission (520 nm). Notably, their quantum yields (QY) differ substantially: DAQx-Bz demonstrates the highest QY (44.6%), followed by DAQx-BP (25.2%) and DAQx-H (7.9%) (Fig. S28). The superior QY of DAQx-Bz can be attributed to its two isolated benzene rings, which introduce additional molecular rotors, enhancing intermolecular interactions in the aggregated state. This restricts non-radiative decay pathways, thereby boosting luminescence efficiency.

**Fig. 5:**
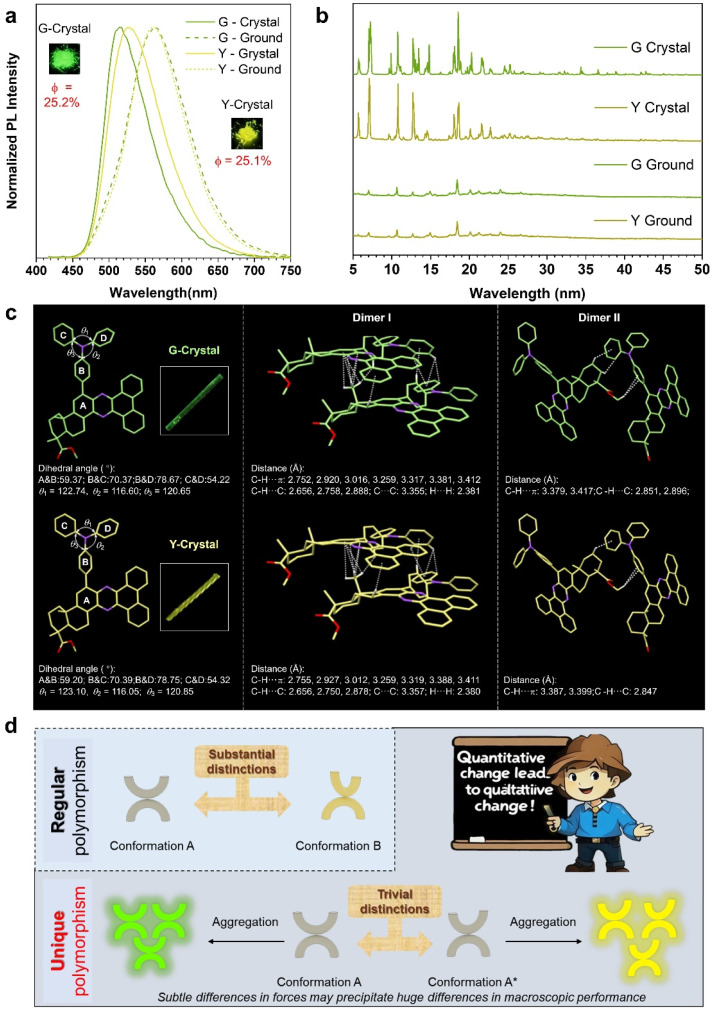
(a) PL spectra and (b) powder X-ray diffraction patterns of DAQx-BP in different crystal states before and after grinding. (c) Crystal structure of DAQx-BP with detailed analysis of molecular conformations and intermolecular interactions as G-crystal and Y-crystal, respectively. (d) Schematic representation of the unique polymorphism with huge different luminescence property based on tiny conformation differences. (A and B represent the dihedral angle of plane A and plane B).

Intriguingly, DAQx-BP exhibits polymorphism-dependent fluorescence (Fig. 5a), yielding two distinct emissive states upon recrystallization from different solvents: green-emitting crystal (G-crystal, *λ*_em_ = 517 nm, QY = 25.2%), and yellow-emitting crystal (Y-crystal, *λ*_em_ = 528 nm, QY = 25.1%). In addition, a third polymorphic state of orange-emitting amorphous (O-pristine, *λ*_em_ = 570 nm, QY = 33.1%) has been obtained as a control (Fig. S29). Through the powder X-ray diffraction (PXRD) spectra, G-crystal and Y-crystal are distinct crystalline phases, evidenced by their sharp diffraction peaks, whereas O-pristine exhibits a broad peak indicative of an amorphous structure (Fig. 5b and S30). In addition, G-crystal and Y-crystal exhibit mechanochromism properties. Upon mechanical grinding, G-crystal and Y-crystal undergo a bathochromic shift in emission, while O-pristine remains unchanged (Fig. S29 and S31). This mechanochromism likely arises from force-induced conformational changes in the D–A framework, altering molecular conjugation, packing, and ICT efficiency. PXRD spectra of ground samples reveal peak broadening and reduced intensity, suggesting a transition toward an amorphous state resembling O-pristine and O-ground (Fig. 5b and S30). Interestingly, O-pristine exhibits a higher QY than its crystalline counterparts (G/Y-crystal), and ground samples (G/Y-ground) show enhanced fluorescence intensity relative to their pristine forms. This contrasts with conventional crystallization-induced emission (CIE) behavior^45^ and may stem from the disruption of long-range π–π stacking, a feature prevalent in G/Y-crystal due to the rigid and conjugated five-membered ring. Mechanical forces likely eliminate these quenching-prone packing motifs, thereby improving emission in the amorphous phase.

To our surprise, single-crystal X-ray diffraction reveals that G-crystal and Y-crystal possess nearly identical molecular conformations and packing arrangements (Fig. 5c, S32, and Table S2), with only minor differences of a twist angle difference of about 0.1° and intermolecular interactions even less than 0.01 Å. Despite these negligible structural deviations, the two polymorphs emit distinct colors. To verify the reliability of the data, we have cultured and tested the single crystal multiple times, and the results confirm the reproducibility of this phenomenon (Fig. S33), underscoring how minute crystalline imperfections can profoundly influence macroscopic luminescent properties. In contrast to regular polymorphism induced by conformations with substantial structural differences, this article has found a unique polymorphism where crystal structures exhibit minimal structural deviations (Fig. 5d). These negligible structural variations in crystalline arise from subtle conformational differences. Yet, when these minute deviations accumulate upon aggregation, they lead to significant differences in macroscopic properties, attributing to the saying of “Quantitative change leads to qualitative change”. This sensitivity to ultra-fine structural modulation highlights the potential of DAQx compounds for high-level anti-counterfeiting technologies.

The reversibility of crystal force-induced color change of these crystals has been investigated. G and Y crystals have been taken and ground on a glass slide; after grinding, both exhibited orange fluorescence. The ground samples are then placed on a heating stage and heated to 150 °C. As the temperature increases, the fluorescence of crystals gradually revert to green (for G) and yellow (for Y). After cooling to room temperature, the crystals fluorescence remains green (for G) and yellow (for Y). This “grinding–heating–cooling” process has been cycled five times, with consistent experimental results (Fig. S34 and S35), confirming that G and Y crystals exhibit reversible force-induced color change behavior. These results demonstrate that the fluorescence red shift to orange of G and Y crystals after mechanical grinding can be restored to their initial states *via* heating, further verifying the reversibility of their force-induced color change properties.

### Dynamic anti-counterfeiting *via* synergistic regulation of polymorphism and acidichromism

**Fig. 6:**
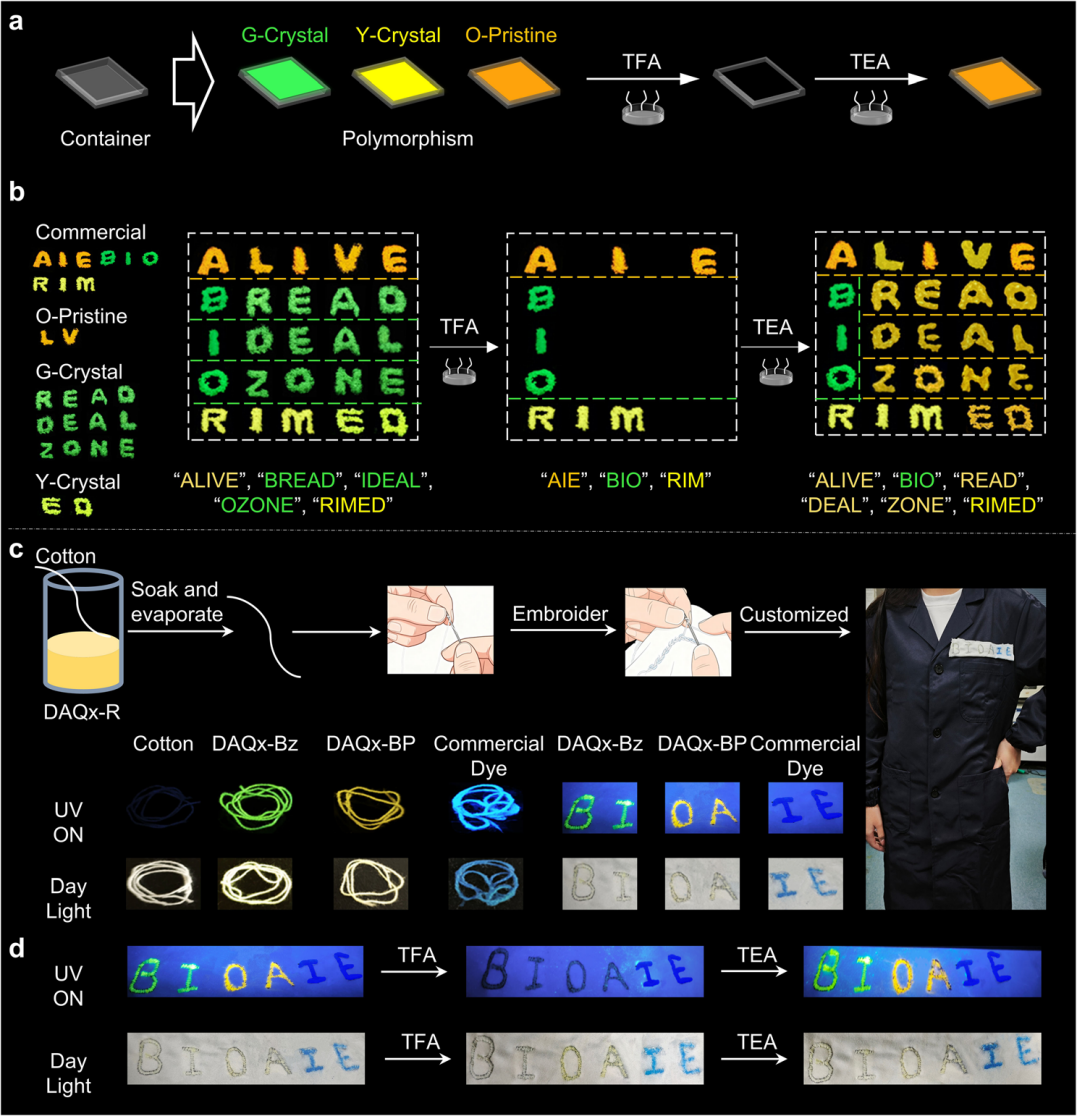
(a) Schematic diagram of polymorphism and acidichromism of DAQx-BP in the solid state. (b) The proof-of-concept demonstrations of encryption–decryption based on DAQx-BP with words mode. (c) Schematic diagram of preparing fluorescent label based on cotton thread dropped with DAQx derivatives. (d) Smart anti-counterfeiting of fluorescent label based on acidichromism.

Beyond its polymorphism-dependent fluorescence, DAQx-BP exhibits pronounced acidichromism, a property enabling reversible fluorescence modulation *via* acid/base vapor exposure. Specifically, the fluorescence of G-crystal, Y-crystal, and O-pristine completely quenches after TFA fuming. Subsequent TEA fuming restores their emission, yielding orange fluorescence (Fig. 6a). Thus, this dual responsiveness (*e.g.*, polymorphism and acidichromism) facilitates multi-mode dynamic encryption–decryption, significantly elevating information security. Here, three distinct encryption modes are demonstrated. First, using binary code for encryption (Fig. S36a), the orange fluorescence samples, such as commercially purchased dyes (like Commercial a), O-pristine, and O-TEA samples, are set to binary code “0”, and the quenched fluorescence samples, such as commercially purchased dyes (like Commercial b) and O-TFA, are set to binary code “1”. Following international binary code rules, the original sequence 01000010-01000001-01000111 is encrypted to “B–A–G”, thus obtaining the information of “bag”. After TFA fuming, the information can be decrypted to as “B–I–O”, while TEA treatment reverts to the original message, demonstrating reconfigurable information storage. Second, a composite of commercial dyes and DAQx-BP polymorphs initially displays four Chinese characters: “
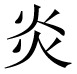
”, “
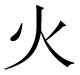
”, “
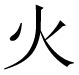
”, and “
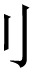
” (Fig. S36b). After TFA fuming, only the Chinese character “
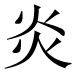
” appears. Then, two Chinese characters, “
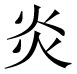
” and “
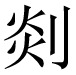
”, appears after TEA fuming, showcasing stimulus-dependent information layering. Finally, modular word encryption is demonstrated (Fig. 6b). The original information is also composed of commercial dyes and polymorphism samples, forming the English words “ALIVE”, “BREAD”, “IDEAL”, “OZONE”, and “RIMED”. Three words, “AIE”, “BIO”, and “RIM”, appears after TFA fuming, and the new words “READ”, “DEAL”, and “ZONE” appears after TEA fuming.

In addition to utilizing the homogeneous polymorphism and acidichromism properties of DAQx-BP to achieve encryption and decryption, DAQx derivatives can also be used for smart anti-counterfeiting. Cotton threads dropped with DAQx-Bz, DAQx-BP, and a commercial blue are embroidered into the label as “BIOAIE” and attracted to clothes (Fig. 6c and d). Under sunlight, “BIOA” appears pale yellow, while “IE” appears blue. Under a 365 nm UV lamp, “BI” emits green light, “OA” emits orange light, and “IE” emits blue light. After fumigating with TFA for 5 minutes under sunlight, “BI” turns white, “OA” turns yellow, and “IE” remains blue; under UV light, “BIOA” fluorescence is quenched, while “IE” remains unchanged. Subsequent fumigation with TEA for 5 minutes restores both color and fluorescence. To evaluate the embroidery material's stability in practical use, the sample is repeatedly washed with laundry detergent, then dried. The fluorescent color remains consistent with the original state, showing no significant attenuation.

Based on these stable optical response characteristics, the system holds potential for smart anti-counterfeiting applications. For instance, preliminary authentication can be performed under daylight and 365 nm UV excitation. If a counterfeit replicates the fluorescent colors, secondary verification can be conducted using daily weak acids/bases (*e.g.*, citric acid, acetic acid, or edible alkali), which enhances security achieves *via* dynamic fluorescence regulation. The smart, responsive fluorescent embroidery strategy demonstrats herein not only expands application pathways in practical scenarios but also provides a feasible solution for next-generation multi-level, high-security anti-counterfeiting technologies.

## Conclusions

The exploration and utilization of natural scaffolds hold significant importance for promoting the innovative development of organic photofunctional materials. In this study, we have successfully designed and synthesized a unique bio-based electron acceptor scaffold, DAQx, derived from renewable rosin resources. By coupling this acceptor with the classical electron donor TPA, we have constructed a series of BioAIE materials with well-defined D–A architectures and systematically investigated the influence of conjugation and ICT on the photophysical properties. Due to the D–A structure, these materials all exhibit solvent-dependent characteristics. Besides, DAQx-BP demonstrates polymorphism-dependent fluorescence, where nearly identical molecular conformations (differing by <0.01 Å in intermolecular interactions) produce distinct green (517 nm) and yellow (528 nm) emission—a rare phenomenon highlighting the extreme sensitivity of solid-state luminescence to subtle packing variations. Furthermore, leveraging these unique properties, multimodal encryption systems operable in both solution and solid states are demonstrated. When DAQx derivatives dropped in nature fiber, it showcases the enormous potential for developing smart anti-counterfeiting materials with acidichromism properties. This work not only introduces DAQx as a new class of natural-product-derived electron acceptors but also provides fundamental insights into polymorphism–fluorescence relationships. It is believed that the BioAIE-based materials will expand the toolbox for sustainable optoelectronic materials and offer new avenues for advanced applications.

It is worth mentioning that DAQx derivatives show a responsiveness to the VOCs. Future studies will focus on elucidating the underlying mechanisms of this material's response behavior to the VOCs. The material holds potential for development into portable detection patches or wearable badges to monitor operators' long-term exposure to VOCs and solvents (*e.g.*, paints, cleaning agents), enabling real-time evaluation of safety exposure limits in industrial or environmental settings”.

## Author contributions

XMC, SFW, JYZ, and BZT conceived and designed the experiments. DMW performed the synthesis. DMW, YTL, XC, LLW, and ZCZ did the PL measurements, QY measurements and analyzed the data; JYZ performed the theoretical calculation. DMW, XC, and XMC co-wrote the paper. WJW, JYZ, ZZ, SLY, and XX took part in the discussion and gave important suggestions.

## Conflicts of interest

There are no conflicts to declare.

## Supplementary Material

SC-017-D5SC09027J-s001

SC-017-D5SC09027J-s002

## Data Availability

CCDC 2443605 and 2443659 contain the supplementary crystallographic data for this paper.^50*a*,*b*^ The data supporting this article have been included as part of the supplementary information (SI). Supplementary information: general experimental details, characterization, additional photophysical properties, DLS, silica gel oxide doping cycling experiment, VOCs sensing capability experiment, different substrate experiments, crystallographic structural analysis, crystal mechanical photochromic cycle experiment. See DOI: https://doi.org/10.1039/d5sc09027j.
