# Supplementary material for: From nature-inspired electron acceptors to BioAIE materials with polarity- and polymorphism-dependence for anti-counterfeiting
Source: Chem Sci. 2025 Dec 15;17(5):2847–57. doi: 10.1039/d5sc09027j (PMC12704454; doi:10.1039/d5sc09027j)
Supplement: SC-017-D5SC09027J-s001 [file SC-017-D5SC09027J-s001.pdf]

Supplementary Information (SI) for Chemical Science.
This journal is © The Royal Society of Chemistry 2025

Supplementary Information

From nature-inspired electron acceptors to BioAIE materials with polarity- and polymorphism-dependence for anti-counterfeiting

Dongmei Wang, [‡a] Xiao Chen [‡ab] Yuting Lin, [‡a] Lulu Wu,[a] Zhichen Zhao,[a] Xu Xu,[a] Shilong Yang,[c] Jianyu Zhang,*[d] Wen-Jin Wang,[b] Zheng Zhao,[b] Shifa Wang,*[a] Ben Zhong Tang,*[b] and Xu-Min Cai*[ae]

[a] Jiangsu Co-Innovation Center of Efficient Processing and Utilization of Forest Resources, College of Chemical Engineering, Nanjing Forestry University, Nanjing 210037, China

[b] Guangdong Basic Research Center of Excellence for Aggregate Science, School of Science and Engineering, The Chinese University of Hong Kong, Shenzhen, Guangdong 518172, China.

[c] Advanced Analysis and Testing Center, Nanjing Forestry University, Nanjing 210037, China

[d] State Key Laboratory of Biobased Transportation Fuel Technology, Department of Polymer Science and Engineering, Zhejiang University, Hangzhou, 3100587 China.

[e] Guangdong Provincial Key Laboratory of Luminescence from Molecular Aggregates, Guangzhou 510640, China

***Corresponding authors**
E-mails:
zhangjianyu@zju.edu.cn;
wangshifa65@163.com;
tangbenz@cuhk.edu.cn;
xumin.cai@njfu.edu.cn

†Equally contributed to this work

Experimental section

Materials and reagents

The natural product dehydroabietic acid (DA), was extracted from commercially disproportionated rosin (Guilin Songquan Forest Chemical Co., Ltd.). Its derivative DAMBDA was synthesized according to the procedures previously reported in the literature and was used as the precursor in this work. Chloroform-d_3 (Aladdin, 99.8%), THF (Aladdin, for spectroscopy, 99.5%), trifluoroacetic acid (TFA) (Energy Chemical, 99%), and triethylamine (TEA) (Sinopharm Chemical Reagent Co., Ltd., 99.0%) were used without further purification. All organic solvents were used as received without further purifications.

Characterization techniques

^{1}H and ^{13}C spectra were carried out on a Bruker AVANCE-III-600 spectrometer (^{1}H, 600 MHz; ^{13}C, 150 MHz) with deuterated chloroform ($CDCl_3$) utilized as the solvent. High-resolution mass spectra (HRMS) were measured on a Q Exactive (Thermo Fisher, Germany) mass spectrometer operating in an ESI mode. Powder X-ray diffraction (PXRD) pattern was acquired utilizing a Rigaku Ultima IV diffractometer with Cu Kα radiation. Ultraviolet-visible (UV-Vis) absorption spectra were collected on a Shimadzu UV2450 spectrometer. Photoluminescence (PL) spectra and absolute fluorescence quantum yields were collected on a Horiba Fluoromax-4 spectrofluorometer. Single crystal data of G-Crystal and Y-Crystal of DAQx-BP were selected and mounted on an XtaLAB Synergy, Dualflex, HyPix diffractometer using Cu Kα radiation (λ = 1.54184 Å). CCDC numbers: 2443605 (for G-Crystal) and 2443659 (for Y-Crystal). Single crystal data of Y-Crystal of DAQx-BP in the second test were selected and mounted on an Xcalibur, Eos, Gemini diffractometer using Mo Kα radiation (λ = 0.71073 Å). Using Olex2, the structure was solved with the ShelXT structure solution program using intrinsic phasing and refined with the ShelXL refinement package using least squares minimization. The ground-state geometries and corresponding frontier molecular orbitals were calculated using the density functional theory (DFT) method at the B3LYP-D3BJ/6-31G(d,p) level.

Extraction and Synthesis

DA: 100 g of powdered disproportionated rosin was placed in a 1000 mL beaker, and 250 mL of 95% ethanol (EtOH) by mass was added. The mixture was heated and stirred until dissolved. At 80 °C, 19.2 mL of excess ethanolamine was added dropwise. After 30 minutes of reaction, 250 mL of preheated deionized water was added. The mixture was extracted with 330 mL of petroleum (PE) ether three times while still hot until the upper layer became colorless. The brownish-yellow aqueous phase was cooled and crystallized, and then filtered to obtain a light yellow solid, which was the crude ethanolamine salt of dehydroabietic acid. The crude product was recrystallized three times with 325 mL of 50% EtOH to obtain white crystals of the ethanolamine salt of dehydroabietic acid. The crystals were dissolved in 325 mL of 50% EtOH, then acidified with 10% hydrochloric acid (HCl) to pH = 4, and 20 mL of distilled water was added for cooling and crystallization. After thorough crystallization, the mixture was filtered, and the filter cake was washed with hot water. Finally, 32 g of pure dehydroabietic acid was obtained.

DAQx-Bz intermediate: A mixture of DAMBDA (synthezied from DA accord to reference[1]) (114.4 mg, 0.3 mmol) and benzil (63.1 mg, 0.3 mmol) in acetic acid (5 mL) is heated to reflux for 12 h. After cooling to room temperature, the mixture is poured into water and the precipitated solid is filtered under reduced pressure to obtain white solid with a yield of 80%. ^{1}H NMR (600 MHz, Chloroform-d_3) 8.00 (s, 1H), 7.58 (dd, J = 36.0, 6.0 Hz, 4H), 7.37–7.31 (m, 5H), 3.69 (s, 3H), 3.63 (dd, J = 18.0, 6.0 Hz, 1H), 3.28–3.22 (m, 1H), 2.38 (dd, J = 33.0, 15.0 Hz, 2H), 1.95–1.88 (m, 1H), 1.84–1.79 (m, 3H), 1.71–1.56 (m, 3H), 1.33 (s, 6H). ^{13}C NMR (150 MHz, Dimethyl sulfoxide-d_6) δ 177.81, 152.35, 152.07, 151.11, 139.77, 138.53, 138.38, 135.99, 132.76, 130.77, 129.79, 129.71, 128.97, 128.15, 128.10, 120.53, 51.95, 47.01, 44.91, 37,59, 37.06, 35.88, 25.41, 23,92, 20.37, 17.97, 16.26. HRMS (ESI, m/z): $[M+H]^+$ calcd for $C_{32}H_{31}BrN_2O_2$: 557.1632; found 557.1625.

DAQx-Bp intermediate: DAQx-BP intermediate is prepared according to the above synthesis procedures of DAQx-Bz intermediate, with 9,10-Phenanthraquinone instead of benzil. ^{1}H NMR (600 MHz, Chloroform-d_3) δ 9.46 (d, J = 6.0 Hz, 1H), 9.34 (d, J = 6.0 Hz, 1H), 8.55 (d, J = 12.0 Hz, 2H), 8.11 (s, 1H), 7.81 – 7.70 (m, 4H), 3.85 (dd, J = 21.0, 9.0 Hz, 1H), 3.73 (s, 3H), 3.48 – 3.41 (m, 1H), 2.44 (t, J = 12.0 Hz, 2H), 2.03 – 1.96 (m, 1H), 1.92 – 1.82 (m, 3H), 1.75 – 1.74 (m, 2H), 1.63 (t, J = 12.1 Hz, 1H), 1.38 (d, J = 6.0 Hz, 6H). ^{13}C NMR (150 MHz, Chloroform-d_3) δ 179.03, 150.56, 141.91, 141.75, 141.48, 138.00, 133.62, 132.33, 132.07, 131.00, 130.45, 130.31, 128.18, 127.97, 126.77, 126.50, 123.07, 122.95, 121.75, 52.25, 47.92, 45.35, 38.17, 37.92, 36.61, 26.09, 24.64, 21.39, 18.68, 16.67. HRMS (ESI, m/z): $[M+H]^+$ calcd for $C_{32}H_{29}BrN_2O_2$: 553.1490; found 553.2587.

DAQx-H: To a solution of DAMBDA (228.8 mg, 0.6 mmol) in 5 mL of EtOH is added 40% glyoxal solution in water (206.0 μL). The mixture is refluxed for 10 hours. The mixture is concentrated in vacuo and the residue is extracted three times with ethyl acetate (EA)/H_2O and washed with saturated $NaHCO_3$ and brine. The obtained mixture is dried with anhydrous sodium sulfate, filtered, rotary evaporated, and purified by silica gel column chromatography to obtain a yellow solid with a yield of 30%. Under a nitrogen atmosphere, a mixture of the obtained yellow solid (80.6 mg, 0.2 mmol), (4-(diphenylamino)phenyl)boronic acid (57.8 mg, 0.2 mmol), and $Pd(PPh_3)_4$ (9.2 mg, 0.008 mmol) is stirred in dry toluene (2 mL). Then, 2M K_2CO_3 (aq) solution (400 μL) is added. The reaction mixture is heated to reflux for 5 h. After cooling, the product is extracted with DCM, washed with H_2O, dried over $MgSO_4$, filtered, concentrated and further purified via column chromatography to afford a pale-yellow solid with a yield of 70%. ^{1}H NMR (600 MHz, Chloroform-d_3) δ 8.83–8.82 (m, 2H), 7.77 (s, 1H), 7.55 (d, J = 12.0 Hz, 2H), 7.30–7.27 (m, 4H), 7.21–7.17 (m, 6H), 7.05 (t, J = 12.0 Hz, 2H), 3.70 (s, 3H), 3.62 (dd, J = 18.0, 6.0 Hz,1H), 3.32–3.25 (m, 1H), 2.50 (d, J = 12.0 Hz,1H), 2.40 (dd, J = 12.0, 6.0 Hz,1H), 2.00–1.92 (m, 1H), 1.89–1.78 (m, 3H), 1.72–1.60 (m, 3H), 1.36 (d, J = 12.0 Hz, 6H). ^{13}C NMR (150 MHz, Chloroform-d_3) δ 179.0,

150.2, 147.8, 147.3, 143.6, 143.3, 142.2, 139.6, 138.0, 132.8, 131.4, 129.4, 127.9, 124.9, 123.2, 122.9, 52.2, 47.9, 45.2, 38.0, 36.6, 26.0, 24.7, 24.6, 21.4, 18.7, 16.7. HRMS (ESI, m/z): $[M+H]^+$ calcd for $C_{38}H_{37}N_3O_2$: 568.2959; found 568.2950.

DAQx-Bz: Under a nitrogen atmosphere, a mixture of the DAQx-Bz intermediate (111.1 mg, 0.2 mmol), (4-(diphenylamino)phenyl)boronic acid (57.8 mg, 0.2 mmol), and $Pd(PPh_3)_4$ (9.2 mg, 0.008 mmol) is stirred in dry toluene (2 mL). Then, 2M K_2CO_3 (aq) solution (400 μL) is added. The reaction mixture is heated to reflux for 5 h. After cooling, the product is extracted with DCM, washed with H_2O, dried over $MgSO_4$, filtered, concentrated and further purified via column chromatography to afford a cyan solid with a yield of 75%. ^{1}H NMR (600 MHz, Chloroform-*d*$_3$) δ 7.82 (s, 1H), 7.74 (d, J = 6.0 Hz, 2H), 7.62 (d, J = 6.0 Hz, 2H), 7.55 (d, J = 6.0 Hz, 2H), 7.36–7.28 (m, 10H), 7.22 (d, J = 12.0 Hz, 6H), 7.06 (t, J = 6.0 Hz, 2H), 3.76–3.72 (m, 4H), 3.40–3.34 (m, 1H), 2.49 (dd, J = 60.0, 12.0 Hz, 2H), 2.01–1.94 (m, 1H), 1.89–1.81 (m, 3H), 1.73–1.66 (m, 3H), 1.39 (d, J = 12.0 Hz, 6H). ^{13}C NMR (150 MHz, Chloroform-*d*$_3$-dmso) δ 179.1, 150.9, 150.9, 150.1, 147.9, 147.2, 139.9, 139.8, 139.5, 137.1, 137.0, 133.0, 132.4, 131.7, 130.2, 130.1, 129.4, 128.7, 128.6, 128.3, 128.2, 127.3, 124.7, 123.1, 123.0, 52.2, 48.0, 45.4, 38.1, 36.6, 26.0, 24.7, 21.5, 18.8, 16.7. HRMS (ESI, m/z): $[M+H]^+$ calcd for $C_{50}H_{45}N_3O_2$: 720.3585; found 720.3589.

DAQx-BP: DAQx-BP is prepared according to the above synthesis procedures of DAQx-Bz. ^{1}H NMR (600 MHz, Chloroform-*d*$_3$) δ 9.38(d, J = 6.0 Hz, 1H), 9.11 (d, J = 6.0 Hz 1H), 8.53–8.51 (m, 2H), 7.90 (s, 1H), 7.84 (d, J = 6.0 Hz, 2H), 7.74–7.70 (m, 3H), 7.64 (t, J = 12.0 Hz, 1H), 7.35–7.32 (m, 6H), 7.29 (t, J = 6.0 Hz, 4H), 7.08 (t, J = 6.0 Hz, 2H), 3.93 (dd, J = 18.0, 6.0 Hz, 1H), 3.75 (s, 3H), 3.57–3.52 (m, 1H), 2.58–2.49 (m, 2H), 2.06–2.03 (m, 1H), 1.92–1.83 (m, 3H), 1.78–1.69 (m, 3H), 1.43 (d, J = 24.0 Hz, 6H). ^{13}C NMR (150 MHz, Chloroform-*d*$_3$) δ 179.3, 151.8, 147.8, 147.7, 141.7, 139.2, 137.7, 134.0, 132.5, 132.1, 131.7, 130.8, 130.3, 130.1, 129.5, 128.8, 127.9, 127.7, 127.1, 127.0, 124.9, 123.4, 122.9, 118.7, 113.8, , 52.5, 47.9, 45.1, 38.1, 37.7, 36.9, 25.6, 24.5, 21.3, 18.6, 16.6. HRMS (ESI, m/z): $[M+H]^+$ calcd for $C_{50}H_{43}N_3O_2$:

718.3428; found 718.3430.

Polarity detection experiment:

First, prepare a stock solution of DAQx-Bp in DCM at a concentration of 1 mg/mL. Subsequently, cut circular filter paper into strips (3 cm × 1 cm × 0.1 mm) and immerse them thoroughly in the prepared stock solution for 30 minutes. After removal, allow the strips to air-dry in a ventilated area to evaporate the DCM solvent, resulting in polar test strips with polarity-responsive functionality. To determine the actual loading of the sample on the filter paper, conduct a sample doping measurement: dissolve DAQx-Bp in DCM to prepare a series of standard solutions at varying concentrations (5, 10, 15, 20, 25, 30, and 35 μmol/L) and perform UV-Vis absorption spectroscopy to establish a standard curve. Additionally, measure the UV absorption values of the DAQx-Bp stock solution before and after immersion of the filter strips, and quantify the sample consumption per filter strip based on the standard curve. Finally, perform polarity detection by applying measured quantities of different polar solvents (e.g., Cy, Et_2O, EA) onto the prepared polar test strips and observe any color changes or other responses.

Smart anti-counterfeiting encryption experiment:

Firstly, dissolve DAQx-Bz and DAQx-Bp solids separately in DCM to prepare two mother solutions with a concentration of 1 mg/mL each. Then, take 300 mg of commercial silica gel and 3 mL of the above mother solutions and mix them thoroughly and stir until evenly distributed. After the DCM solvent has completely evaporated, two silica gel with solvent color-changing properties can be obtained. To achieve image encryption and decryption, an organic solvent fumigation treatment is carried out first: place 20 mg of silica gel in a black circular small cover with a diameter of 1.5 cm, put it in a petri dish containing the specified solvent (such as Cy, Et_2O, EA, etc.), and cover the petri dish with a glass cover for fumigation. Thus, silica gel presenting different fluorescence colors can be obtained. Next, the image preparation and encryption

operation is carried out: after fumigating the silica gel doped with DAQx-Bz and DAQx-Bp with Cy, Et_2O, THF, and DCM respectively, a series of silica gel particles with different fluorescence colors can be obtained. Arrange and combine these silica gel in a flower pattern according to the preset specific sequence. After the solvent has completely evaporated, conduct Cy fumigation treatment on all the silica gel at this time, so that the pattern information is "encrypted" due to the uniform fluorescence color; then, adjust the position of the silica powder and wait for the Cy to completely evaporate, and then fumigate all the silica powders with DCM to "decrypt" and reproduce the preset flower pattern.

Different substrate experiments:

(1) Gold sheet: DQAx-Bp was dissolved in DCM to prepare a stock solution with a concentration of 1 mg/mL. 100 μL of this solution was dropped onto the surface of the gold sheet and the gold sheet was placed on the spin-coating instrument of the uniform coating machine for spinning for 1 minute. This operation was repeated three times to evenly load the DAQx-Bp sample onto the surface of the gold sheet. Then, the gold sheet with the loaded sample was transferred to a glass slide and placed above a petri dish containing the specified solvents (EA, Cy, Et_2O and DCM). A glass cover was invertedly placed over the petri dish to perform solvent fumigation treatment.

(2) PMMA film: Distribute DAQx-Bp into DCM to prepare a stock solution with a concentration of 1 mg/mL; then, dissolve 2.5 g of polymethyl methacrylate (PMMA) in 20 mL of chloroform at 50 °C. After complete dissolution, add 1 mL of the aforementioned DAQx-Bp solution and stir thoroughly to evenly disperse the sample in the PMMA solution. Pour the mixed solution into a circular glass mold with a diameter of 10 cm. Let it stand at room temperature overnight until the solvent completely evaporates, then obtain the dry PMMA film. Cut the film into uniform square pieces (1.5 cm × 1.5 cm × 0.2 mm), and transfer it to a glass slide. Place it above a petri dish containing the specified solvents (EA, Cy, Et_2O, and DCM), cover the petri dish with a glass cover, and perform solvent fumigation treatment.

(3) Cotton thread: DQAx-Bp was dissolved in DCM to prepare a stock solution with a concentration of 1 mg/mL. The white cotton thread was fully immersed in this solution to ensure that the sample was evenly loaded on the surface of the cotton thread. Then, the cotton thread was removed and placed in a ventilated area, allowing the solvent to evaporate completely and dry naturally. The dried cotton thread was cut into short lines of uniform length (2 cm), and then transferred to a slide and placed above a petri dish containing the specified solvents (EA, Cy, Et_2O and DCM). A glass cover was inverted and placed over the petri dish to conduct solvent fumigation treatment.

(4) Cotton thread acid-base stimulus response: Firstly, DAQx-Bz, DAQx-Bp and commercial blue dye were dissolved in DCM respectively to prepare stock solutions with a concentration of 1 mg/mL. Then, the white cotton threads were completely immersed in the above three solutions to ensure that the samples were evenly loaded on the surface of the cotton thread fibers. After that, the cotton threads were taken out and placed in a fume hood to allow the solvents to naturally evaporate until they were completely dry. A piece of white cotton cloth was used as the base, and the cotton threads that were dried and loaded with DAQx-Bz, DAQx-Bp and commercial blue dye were respectively embroidered into the characters "BI", "OA" and "IE", forming the pattern "BIOAIE". The embroidered cotton cloth was placed above the petri dish containing TFA, and a glass cover was inverted to cover it for acidic gas fumigation. After five minutes, the cotton cloth was taken out and placed in the fume hood again, allowing the TFA gas to completely evaporate. Then, it was placed above the petri dish containing TEA with a glass cover inverted to undergo alkaline gas fumigation.

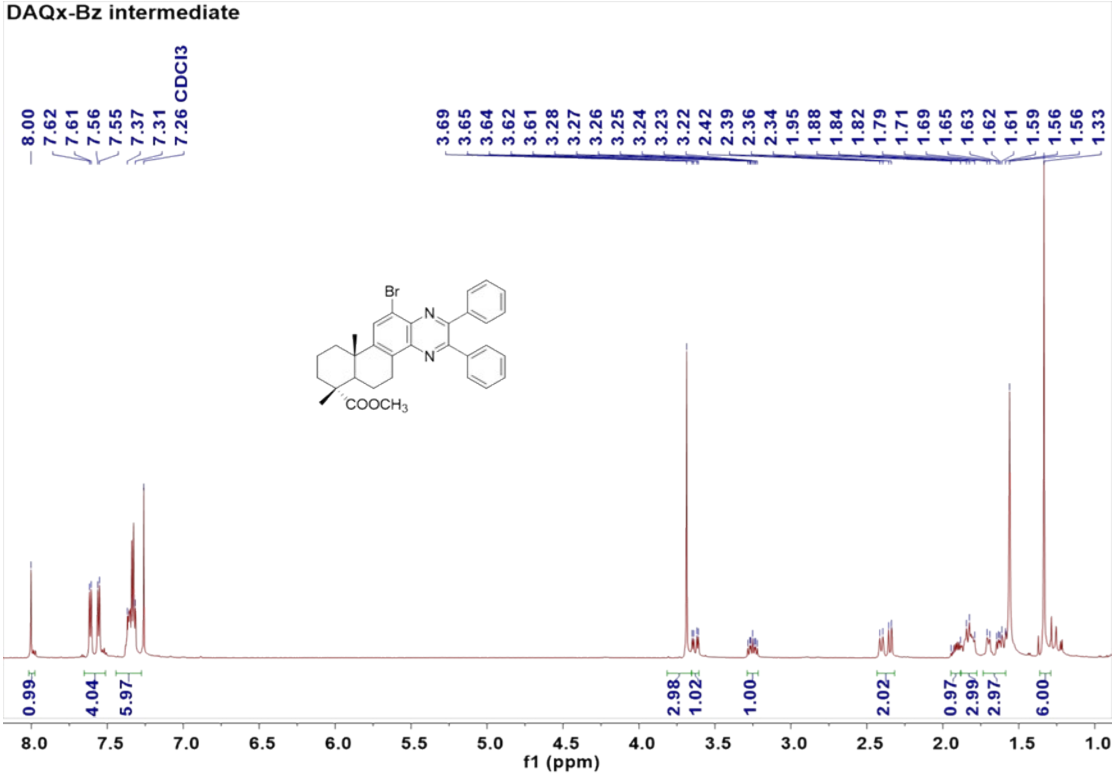

Figure S1. ^{1}H NMR spectrum of DAQx-Bz intermediate in $CDCl_3$-d_3.

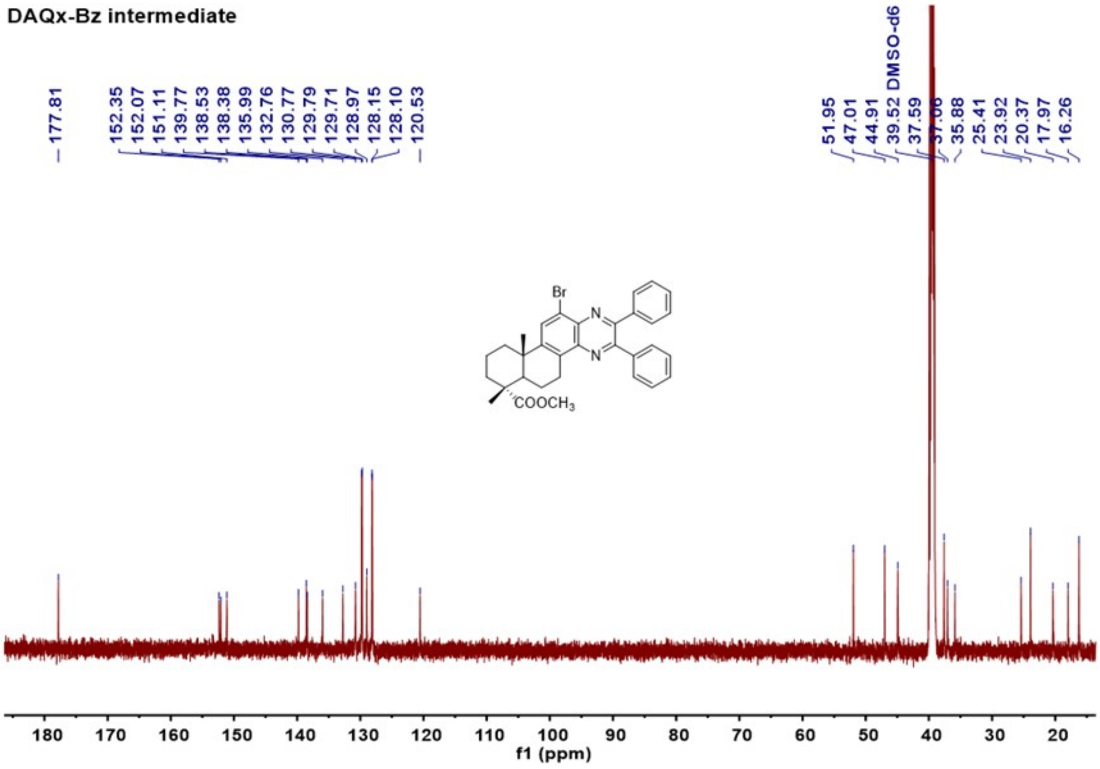

Figure S2. ^{13}C NMR spectrum of DAQx-Bz intermediate in DMSO-d_6.

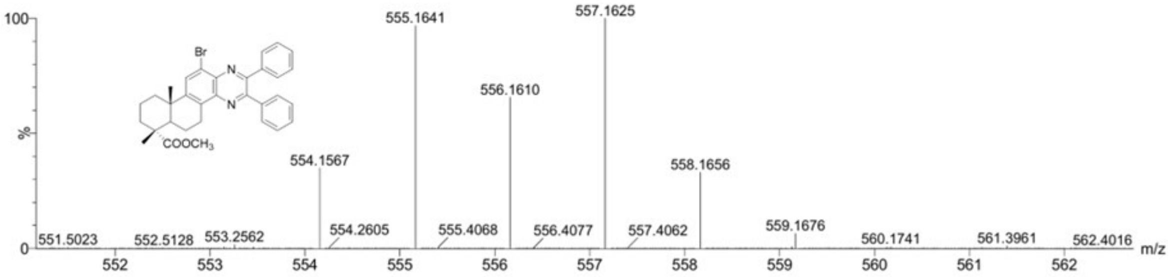

Figure S3. HRMS spectrum of DAQx-Bz intermediate.

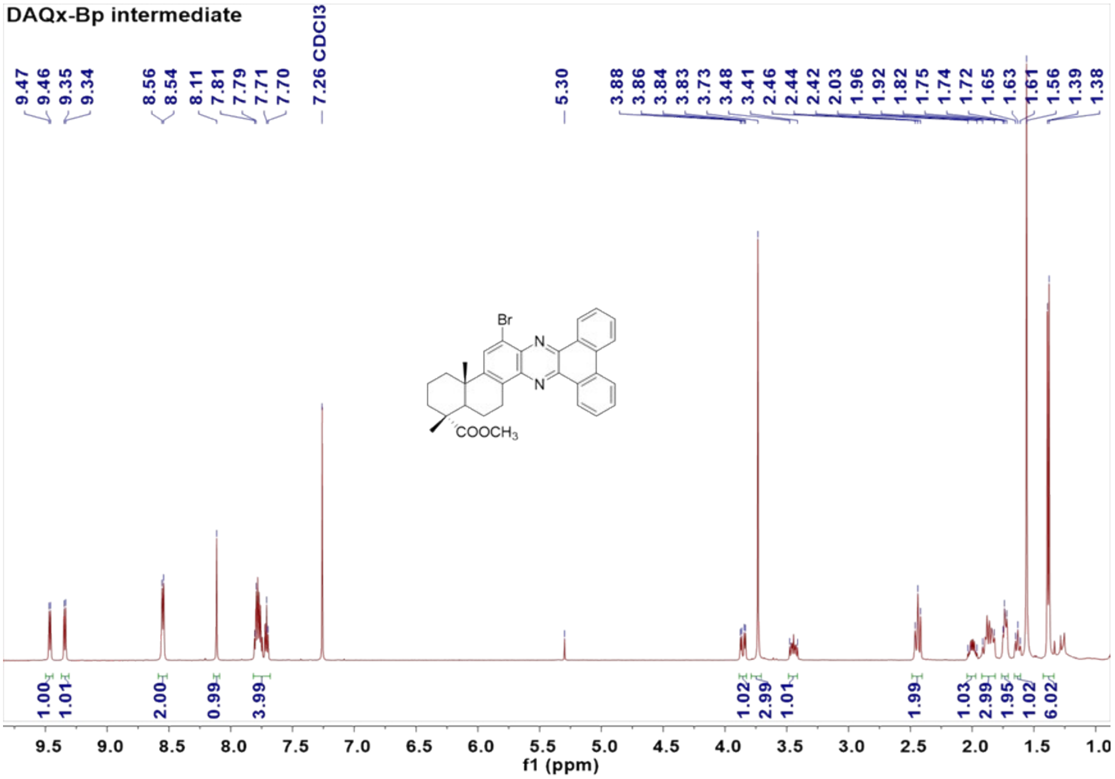

Figure S4. ^{1}H NMR spectrum of DAQx-Bp intermediate in $CDCl_3$-d_3.

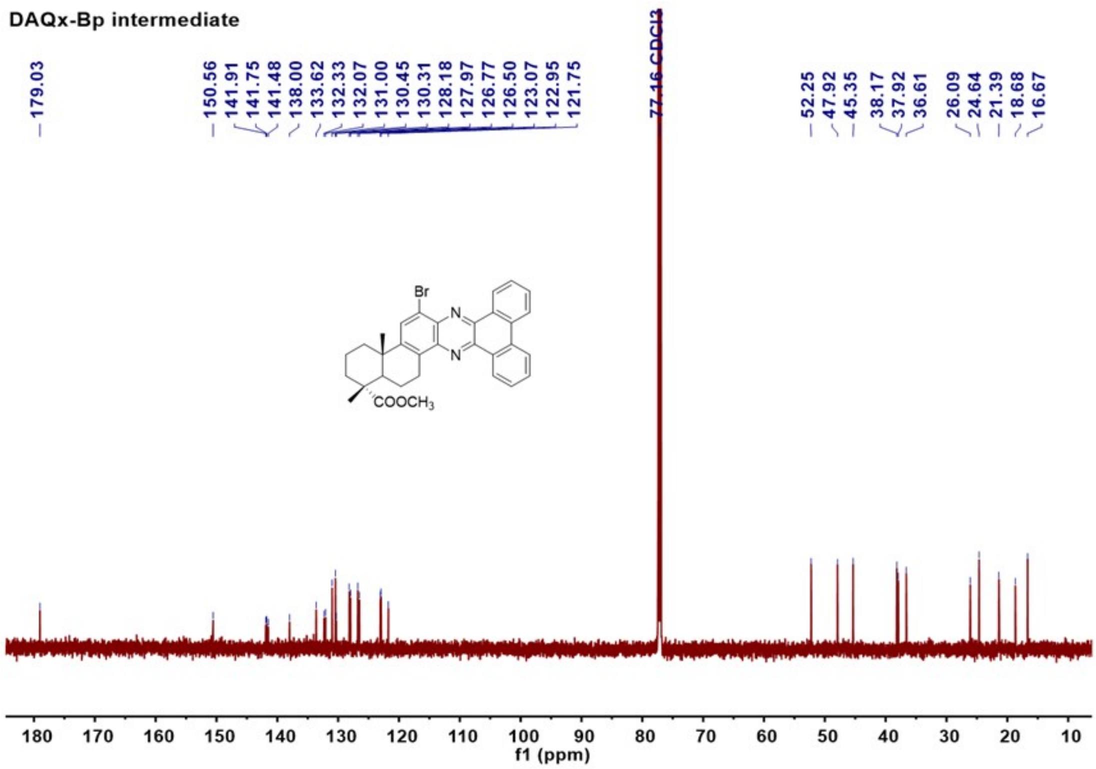

Figure S5. ^{13}C NMR spectrum of DAQx-Bp intermediate in $CDCl_3$-d_3.

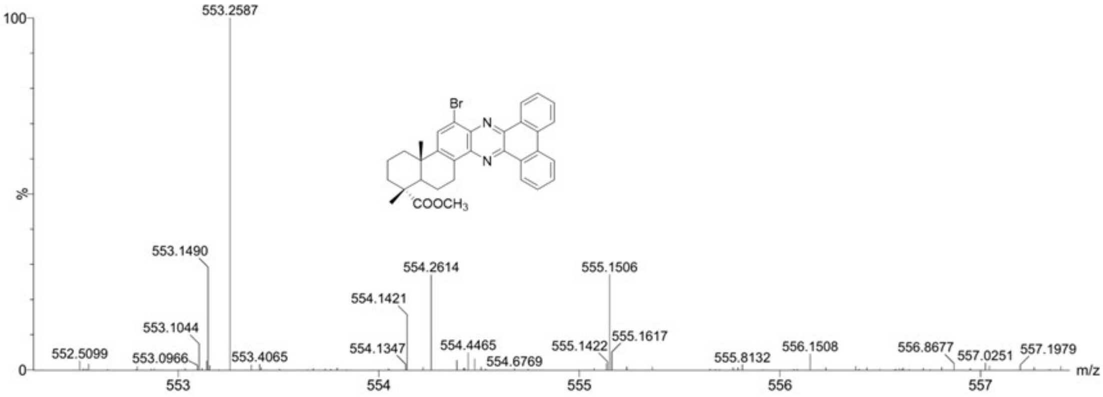

Figure S6. HRMS spectrum of DAQx-Bp intermediate.

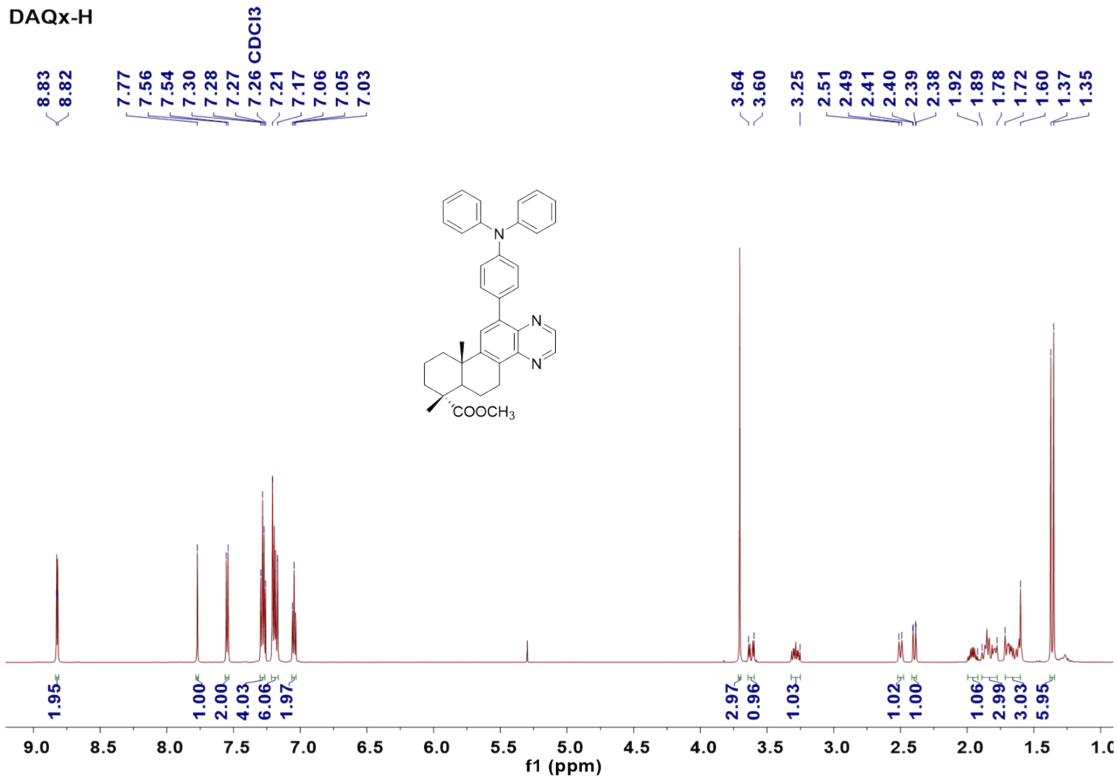

Figure S7. ^{1}H NMR spectrum of DAQx-H in $CDCl_3$-d_3.

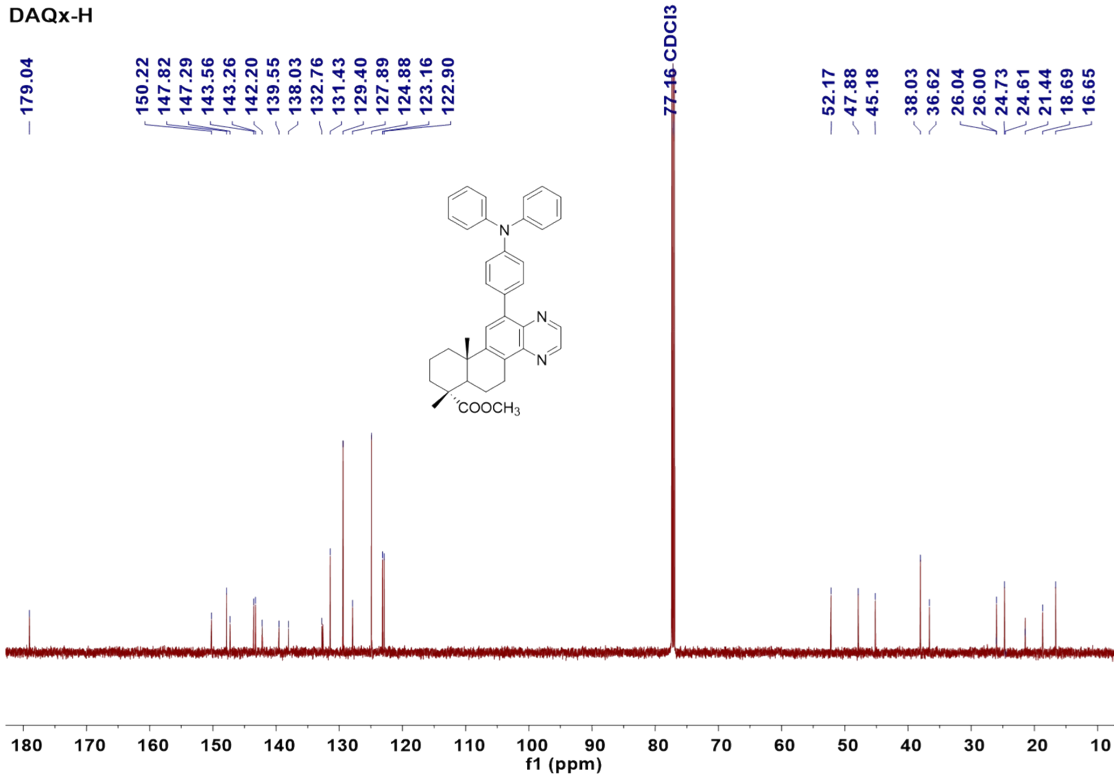

Figure S8. ^{13}C NMR spectrum of DAQx-H in $CDCl_3$-d_3.

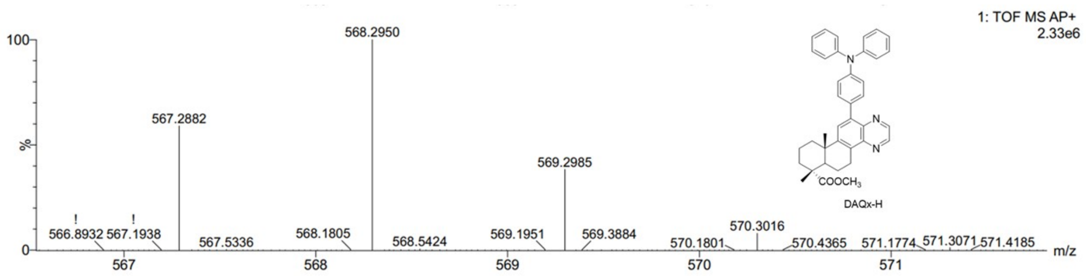

Figure S9. HRMS spectrum of DAQx-H.

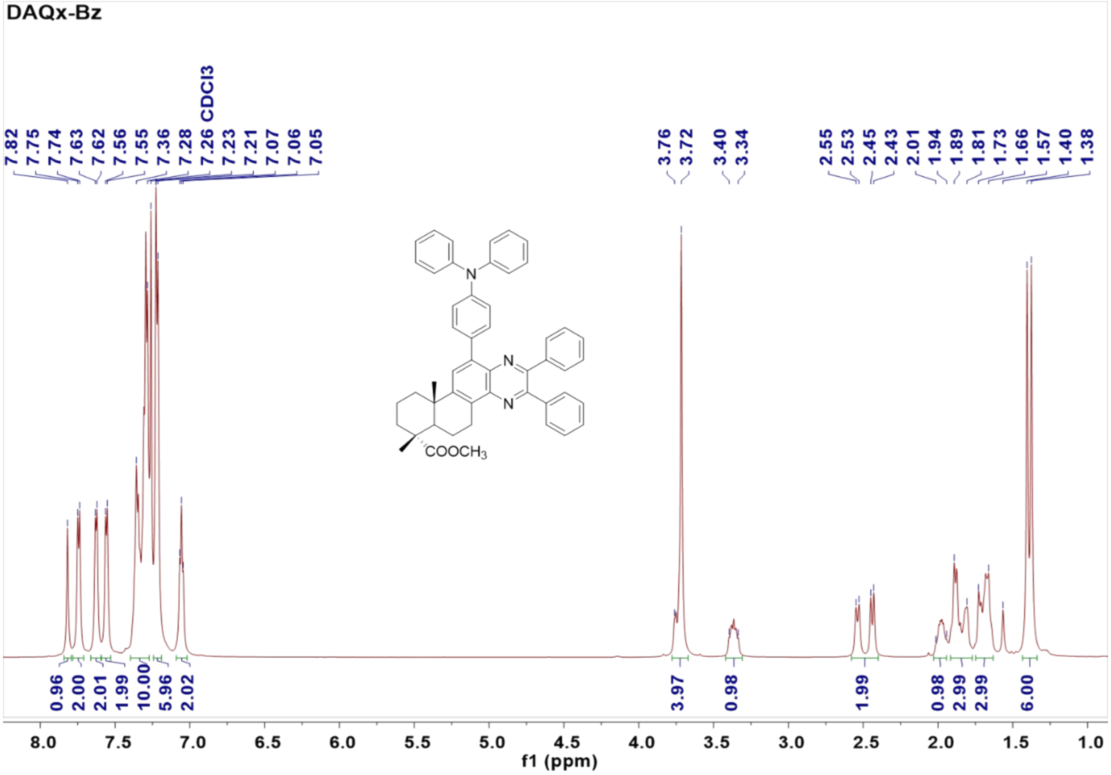

Figure S10. ^{1}H NMR spectrum of DAQx-Bz in $CDCl_3$-d_3.

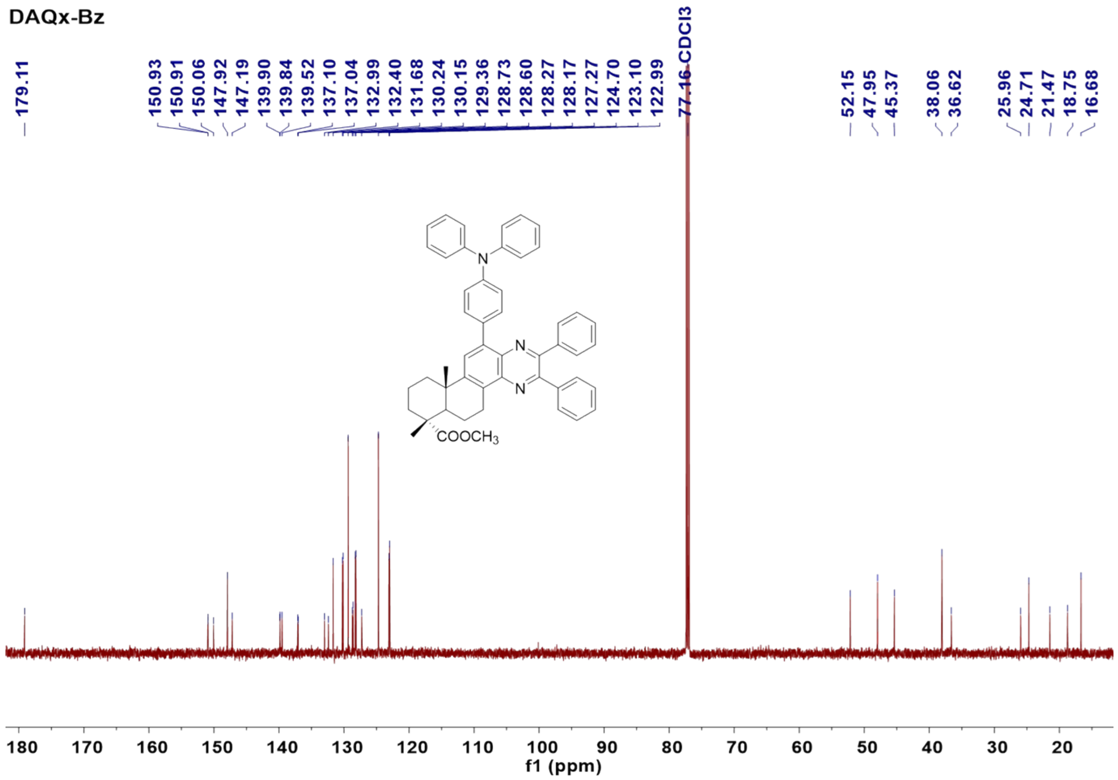

Figure S11. ^{13}C NMR spectrum of DAQx-Bz in $CDCl_3$-d_3.

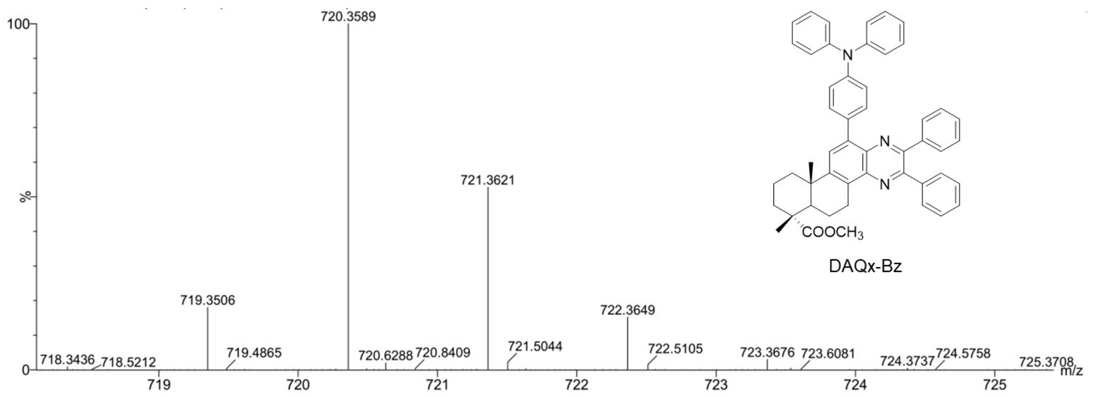

Figure S12. HRMS spectrum of DAQx-Bz.

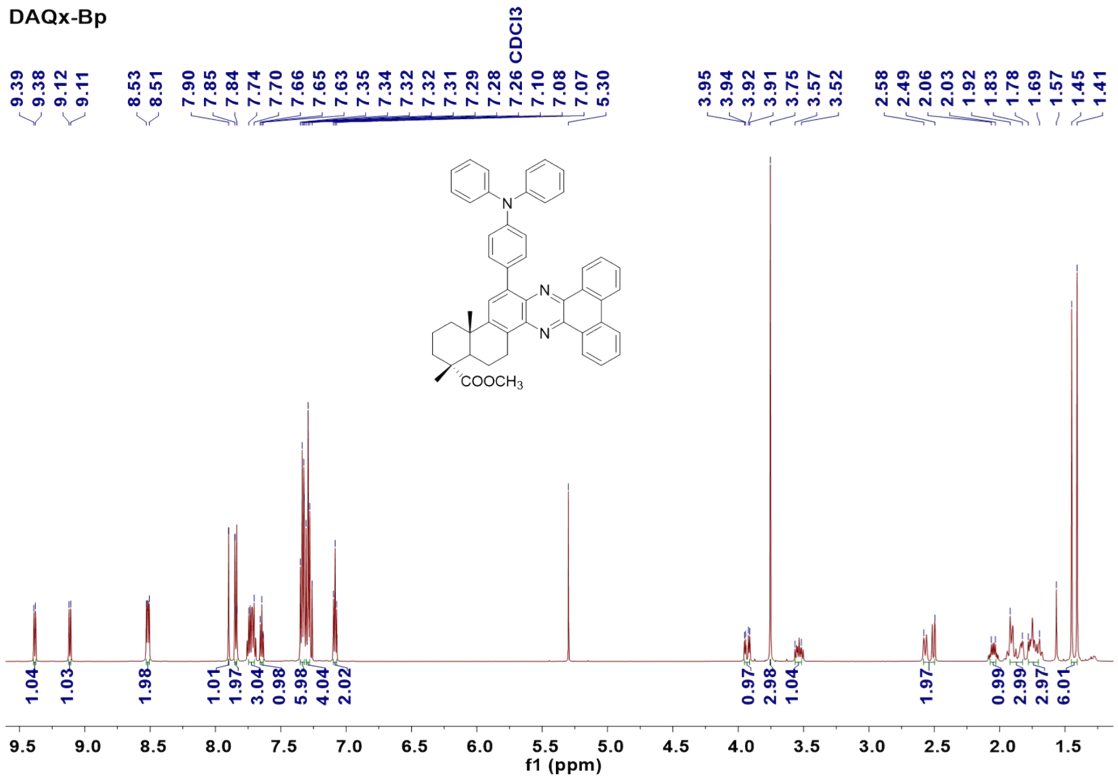

Figure S13. ^{1}H NMR spectrum of DAQx-BP in $CDCl_3$-d_3.

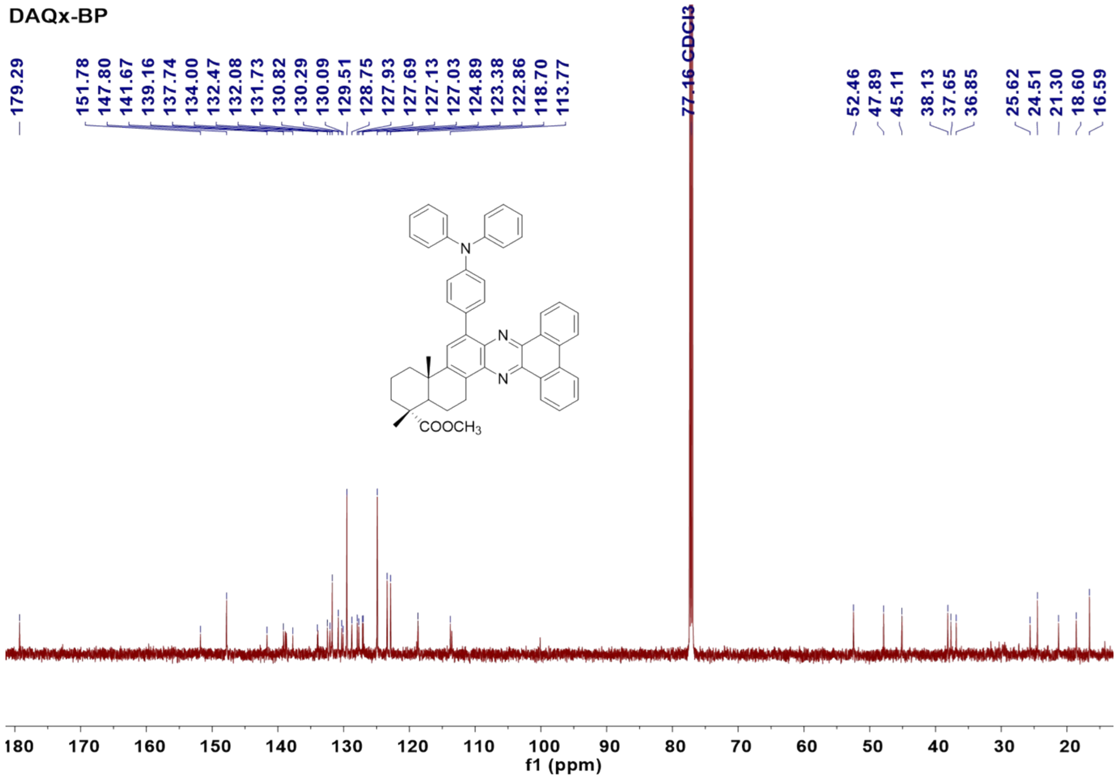

Figure S14. ^{13}C NMR spectrum of DAQx-BP in $CDCl_3$-d_3.

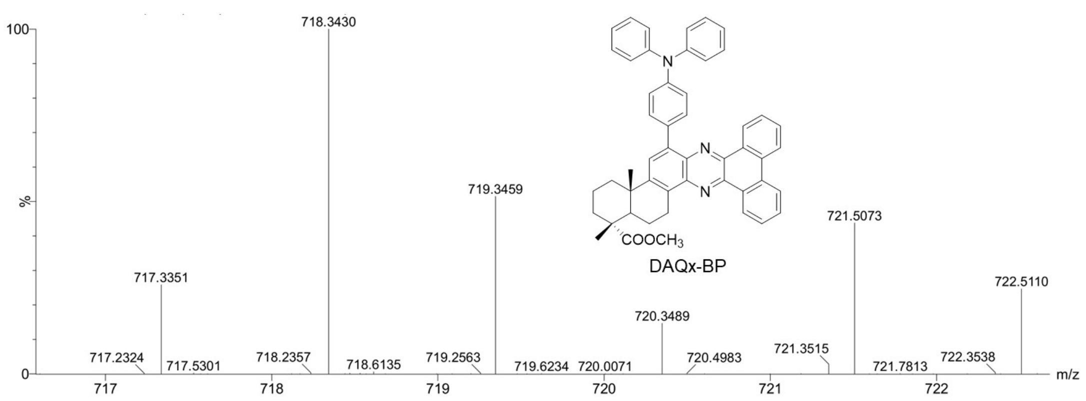

Figure S15. HRMS spectrum of DAQx-BP.

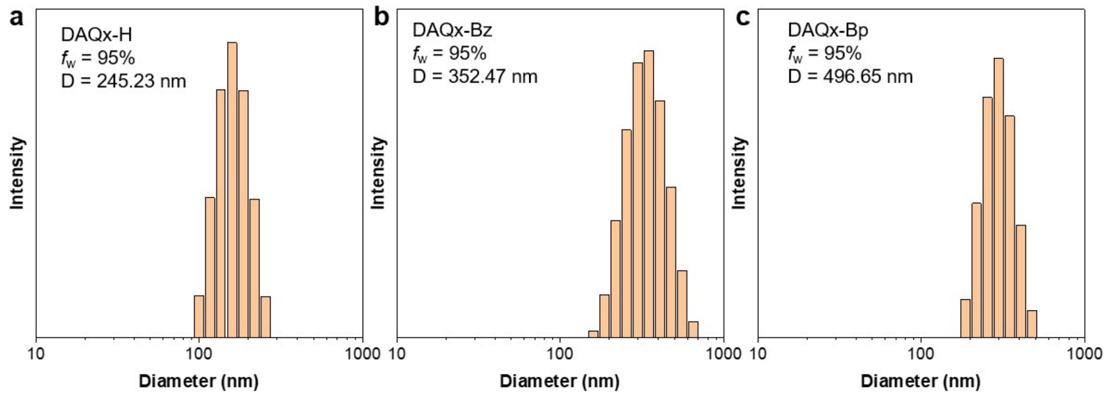

Figure S16. (a) DAQx-H (b) DAQx-Bz and (c) DAQx-Bp with in THF/H_2O mixtures with 95% water fractions (f_w).

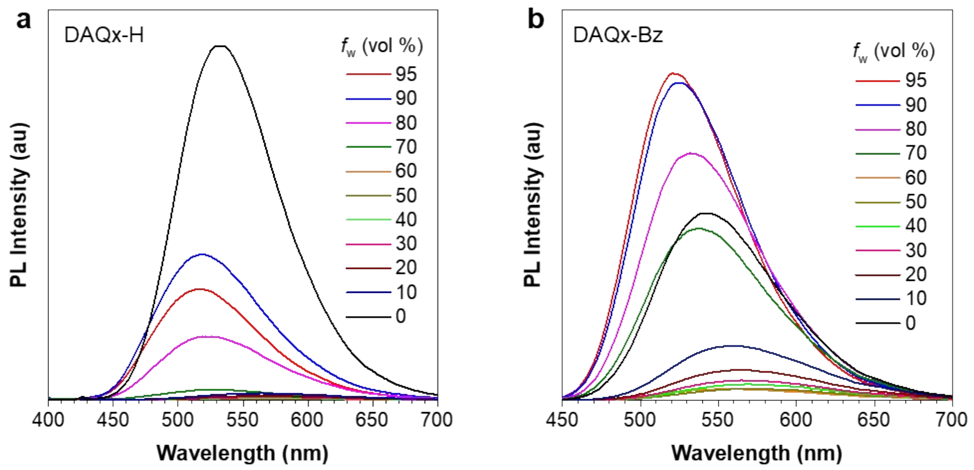

Figure S17. Photoluminescence spectra of (a) DAQx-H and (b) DAQx-Bz in THF/H_2O mixtures with different water fractions (f_w).

Table S1. DAQx-H, DAQx-Bz and DAQx-Bp in THF/H_2O mixtures with different f_w.

	Diameter (nm)	
	f_w = 0%	f_w = 95%
DAQx-H	–	245.23
DAQx-Bz	–	352.47
DAQx-Bp	–	496.65

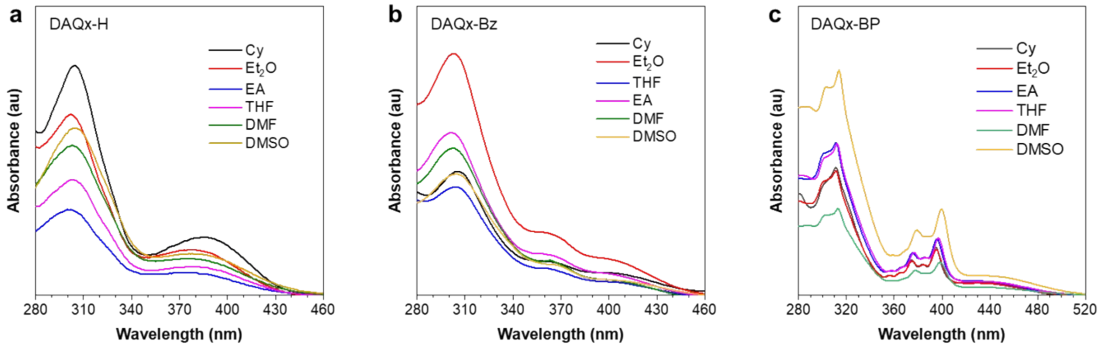

Figure S18. Absorption spectra of (a) DAQx-H, (b) DAQx-Bz, and (c) DAQx-BP in different solvents.

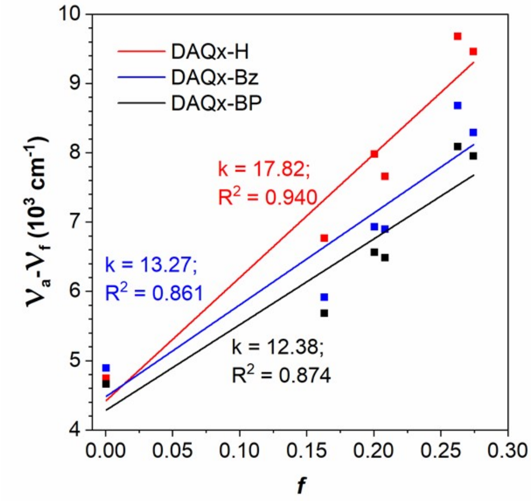

Figure S19. Lippert-Mataga equations of DAQx-H, DAQx-Bz, and DAQx-BP. Linear correlation of orientation polarization (*f*) of solvent media with the Stokes shift (ν_a-ν_f). The *k* value was the slope of the fitting curve.

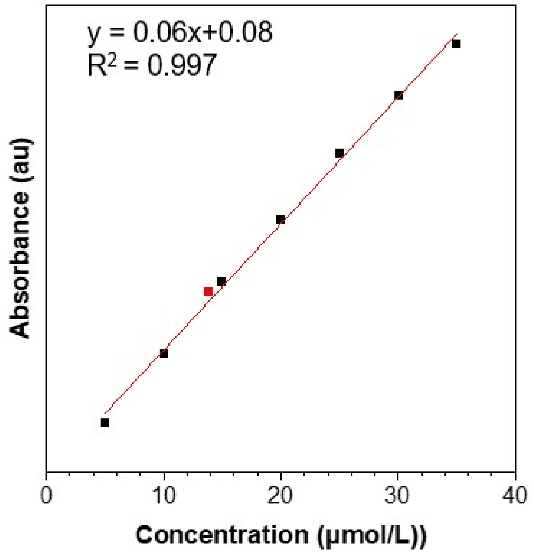

Figure S20. Standard curve based on UV-Vis spectra of DAQx-Bp in DCM.

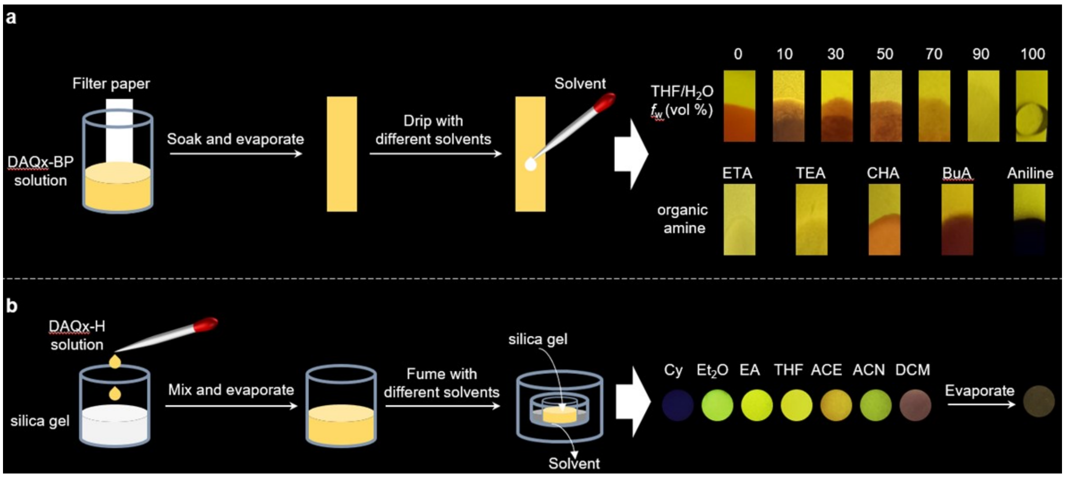

Figure S21. (a) Schematic diagram of fabricating DAQx-BP treated paper and photographs of corresponding fluorescence changing from yellow to multicolor in response to the proportion of solvent mixture (THF/H_2O) and organic amine. (b) Schematic progress of fabricating silica gel powder mixed with DAQx-H response to the varying solvents.

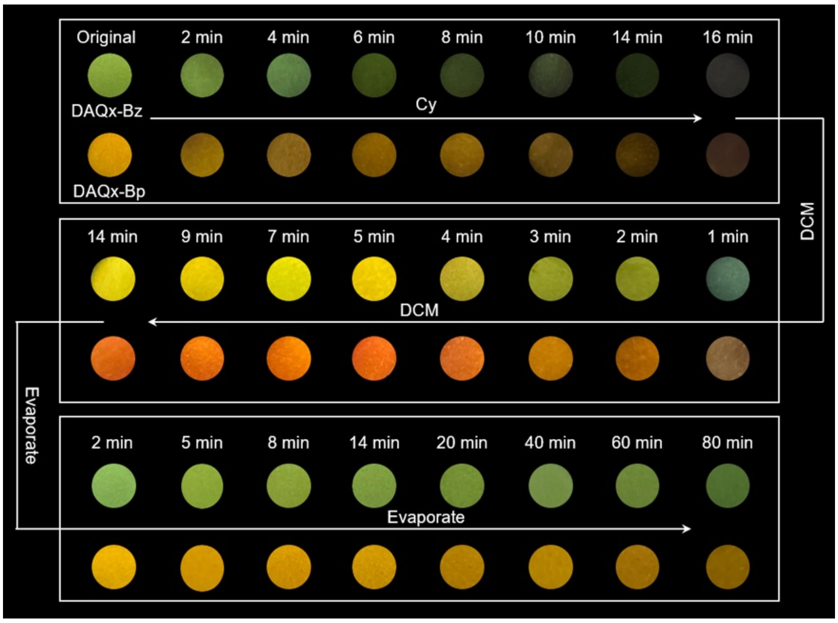

Figure S22. Schematic diagram of the fumigation experiment process using DCM and Cy on the silica gel doped with DAQx-Bz and DAQx-Bp.

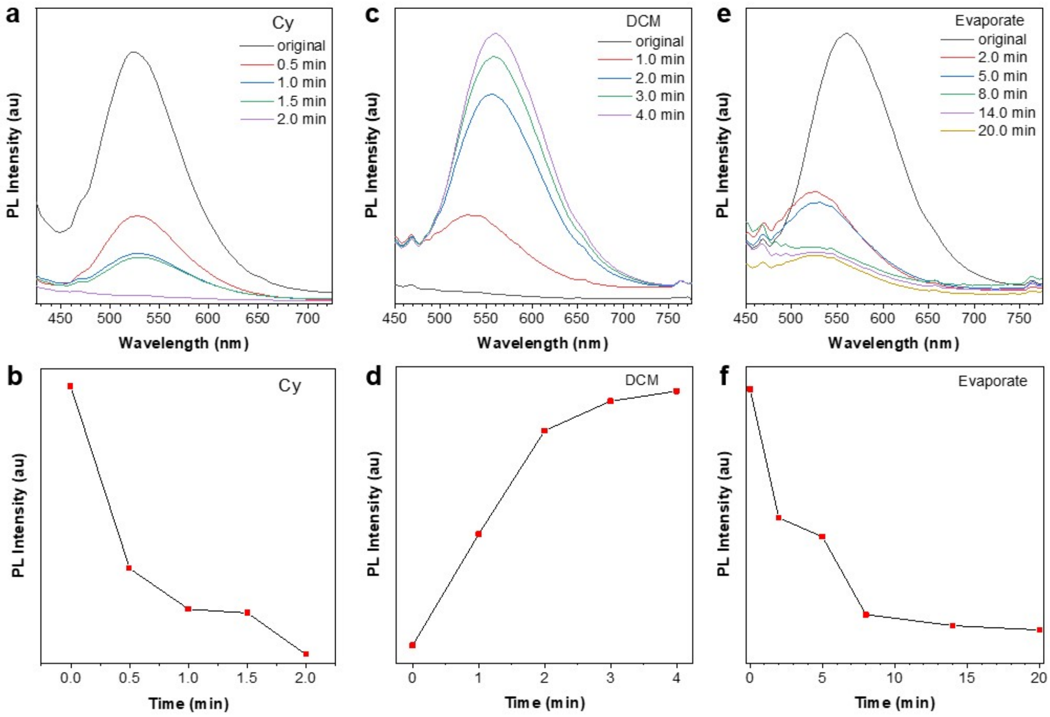

Figure S23. (a) PL spectra and (b) point-line diagrams of silica gel doped with DAQx-Bz after being fumigated with Cy. (c) PL spectra and (d) point-line diagrams of silica gel doped with DAQx-Bz after being fumigated with DCM. (e) PL spectra and (f) point-line diagrams of silica gel doped with DAQx-Bz after being fumigated with DCM and solvent evaporation.

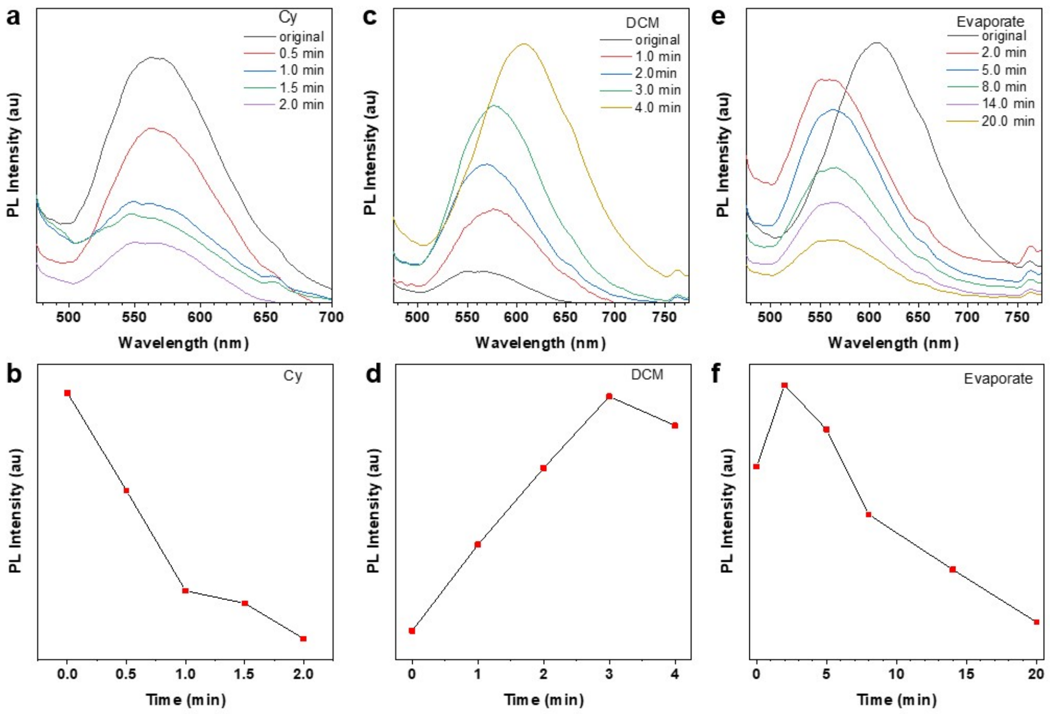

Figure S24. (a) PL spectra and (b) point-line diagrams of silica gel doped with DAQx-Bp after being fumigated with Cy. (c) PL spectra and (d) point-line diagrams of silica gel doped with DAQx-Bp after being fumigated with DCM. (e) PL spectra and (f) point-line diagrams of silica gel doped with DAQx-Bp after being fumigated with DCM and solvent evaporation.

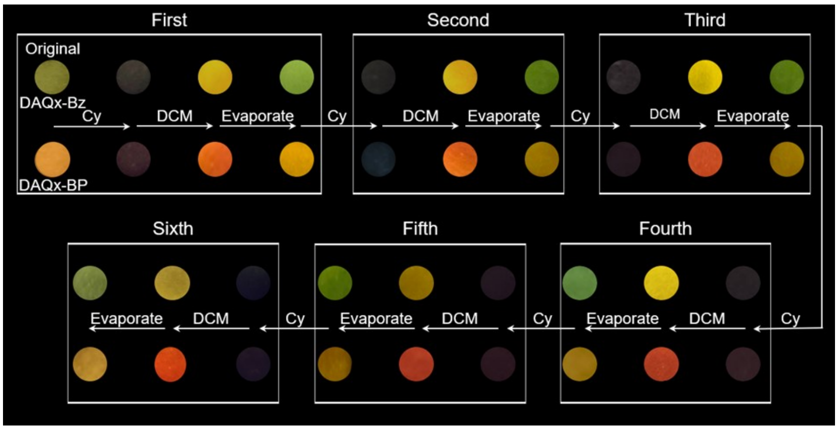

Figure S25. Schematic diagram of the repeated fumigation experiments of silica gel doped with DAQx-Bz and DAQx-Bp using DCM and Cy.

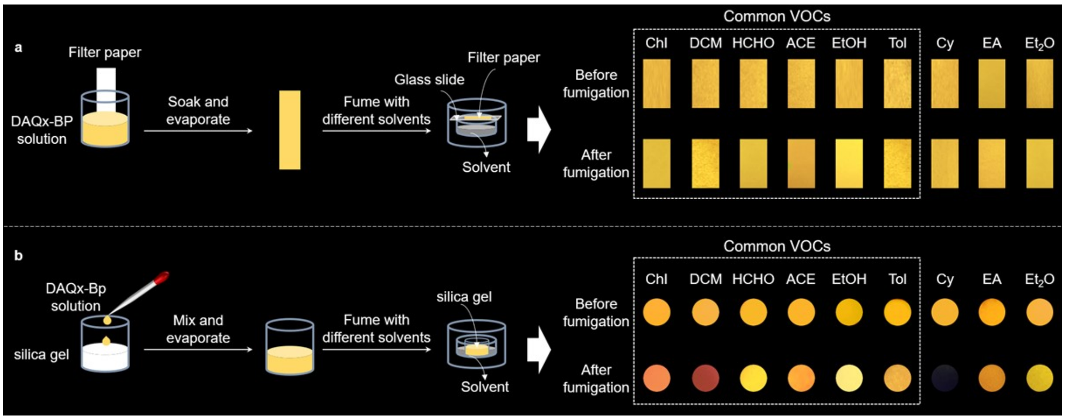

Figure S26. (a) Schematic diagram of the experiment for fumigating silica gel doped with DAQx-Bp with different solvents. (b) Schematic diagram of the experiment using different solvents to fumigate the filter strips loaded with DAQx-Bp.

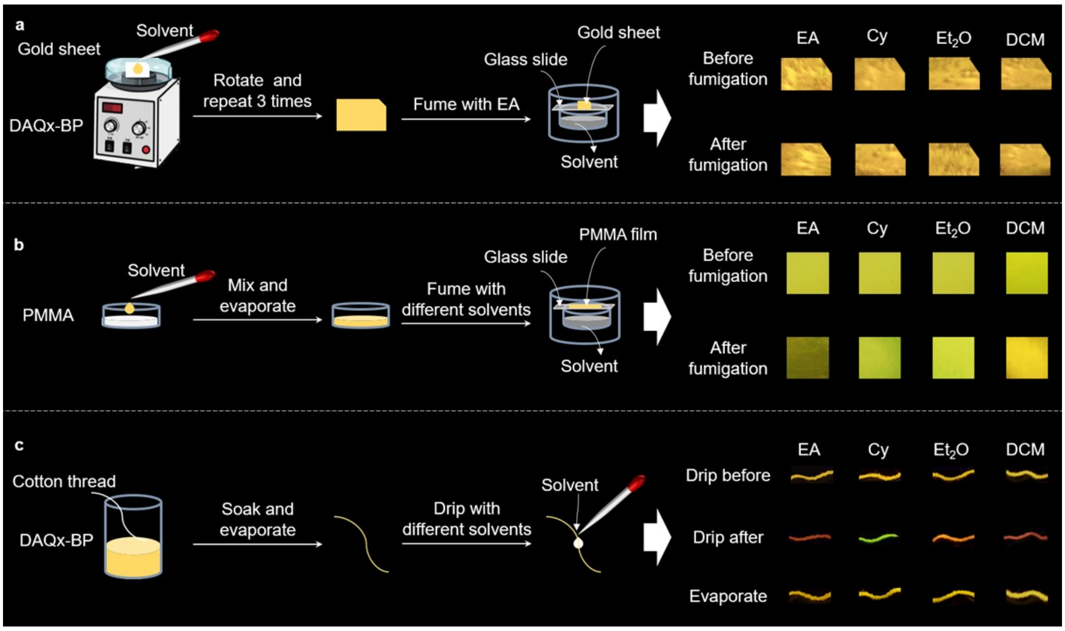

Figure S27. (a) Schematic diagram of solvent vaporization of DAQx-Bp solution on a gold sheet. (b) Schematic diagram of solvent vaporization of DAQx-Bp solution on Polymethyl methacrylate (PMMA) film. (c) Schematic diagram of solvent drop addition of DAQx-Bp solution onto cotton thread.

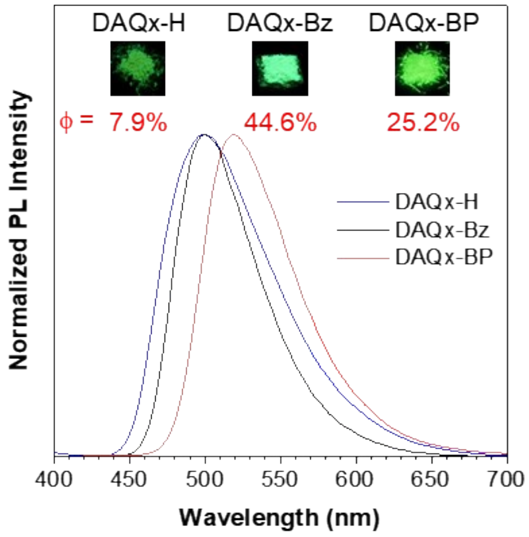

Figure S28. Photoluminescence (PL) spectra and absolute quantum yields of DAQx-H, DAQx-Bz, and DAQx-BP as solid.

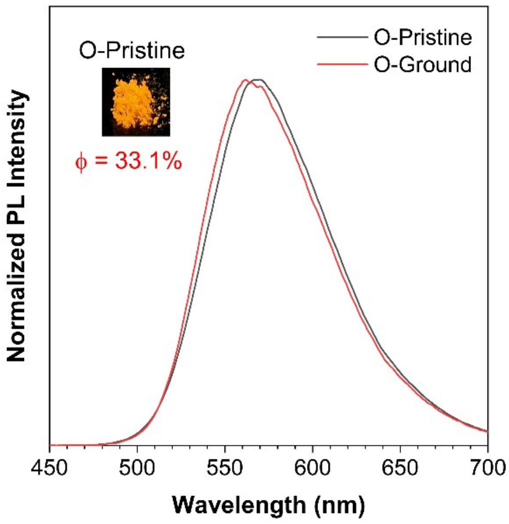

Figure S29. Photoluminescence (PL) spectra of DAQx-BP in ptistine state before and after grinding.

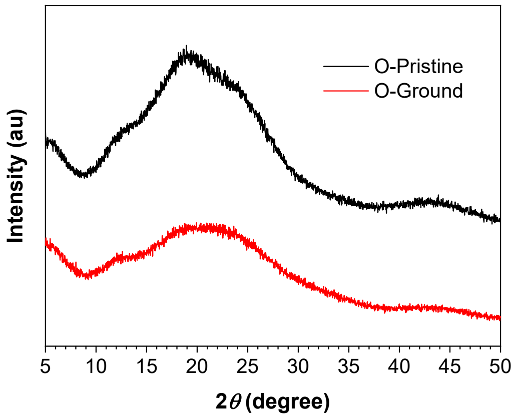

Figure S30. PXRD spectra of (a) G-Crystal, (b) Y-Crystal, and (c) O-Pristine of DAQx-BP before and after grinding.

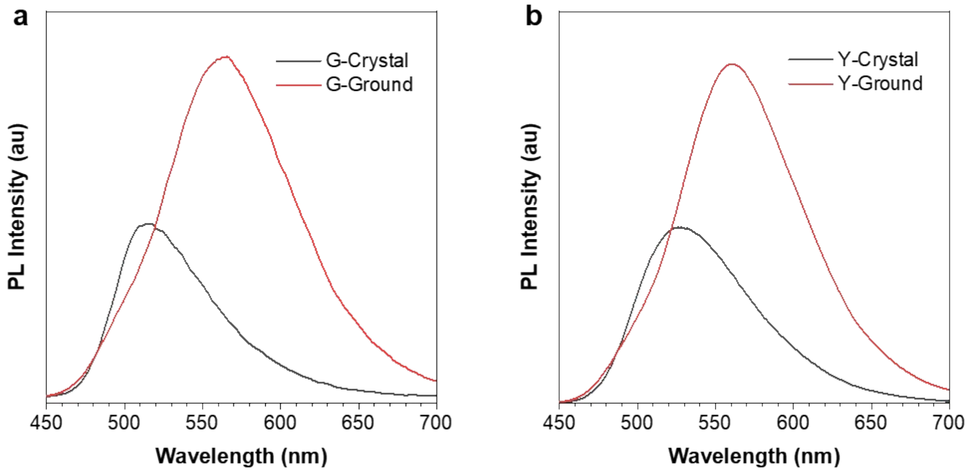

Figure S31. Photoluminescence spectra of (a) G-Crystal and (b) Y-Crystal of DAQx-BP before and after grinding.

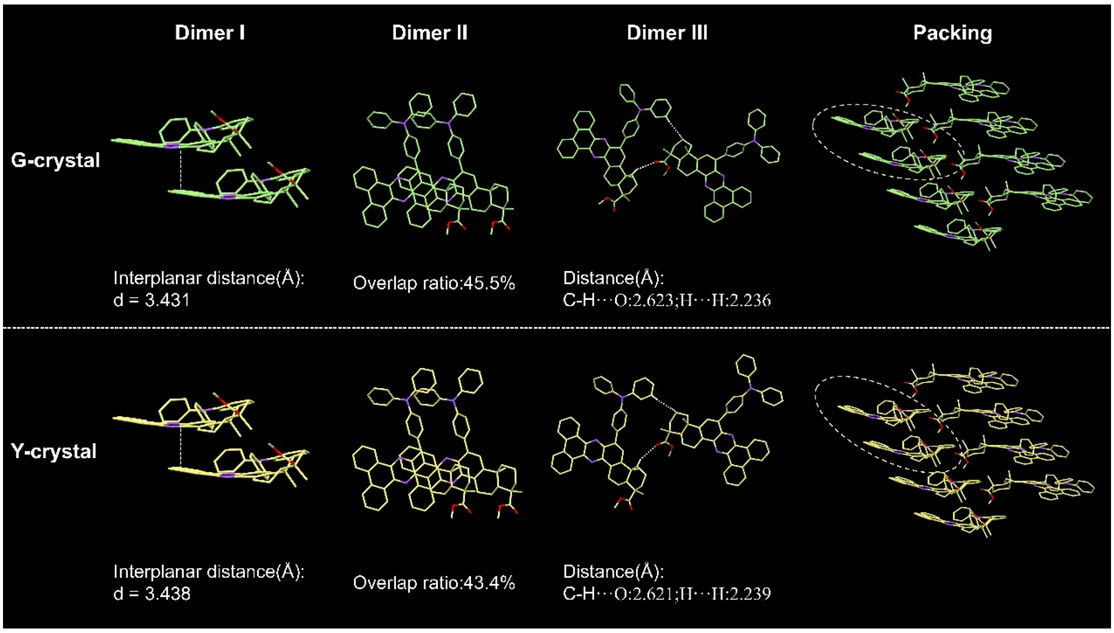

Figure S32. Crystal packing of G-Crystal and Y-Crystal of DAQx-BP.

Table S2. Crystallographic data for G-Crystal and Y-Crystal of DAQx-BP.

	G-Crystal	Y-Crystal	Y-Crystal-2
empirical formula	$C_{50}H_{43}N_3O_2$	$C_{50}H_{43}N_3O_2$	$C_{50}H_{43}N_3O_2$
M_r	717.87	717.87	717.87
cryst syst	orthorhombic	orthorhombic	orthorhombic
space group	$P2_12_12_1$	$P2_12_12_1$	$P2_12_12_1$
a (Å)	6.34630(10)	6.34350(10)	6.3491(3)
b (Å)	16.7092(4)	16.7303(3)	16.7318(9)
c (Å)	36.0686(10)	36.0895(7)	36.113(3)
α (°)	90	90	90
β (°)	90	90	90
γ (°)	90	90	90
V (Å^3)	3824.77(15)	3830.13(12)	3836.4(4)
Z	4	4	4
ρ_c (g cm^{-3})	1.247	1.245	1.243
F(000)	1520	1520	1520
T (K)	293	293.15	293(2)
μ (mm^{-1})	0.591	0.590	0.076
data / restraints / parameters	7663/0/499	7639/0/499	8126/0/499
GOF (F^2)	1.006	1.025	1.015
R_1[a], wR_2[b] ($I > 2\sigma(I)$)	0.0496, 0.1179	0.0450, 0.1117	0.0741, 0.0950
R_{int}	0.0937	0.0749	0.0731

[a] $R_1 = \Sigma(||F_o| - |F_c||)/\Sigma|F_o|$; [b] $wR_2 = \{\Sigma[w(F_o^2 - F_c^2)^2]/\Sigma[w(F_o^2)^2]\}^{1/2}$

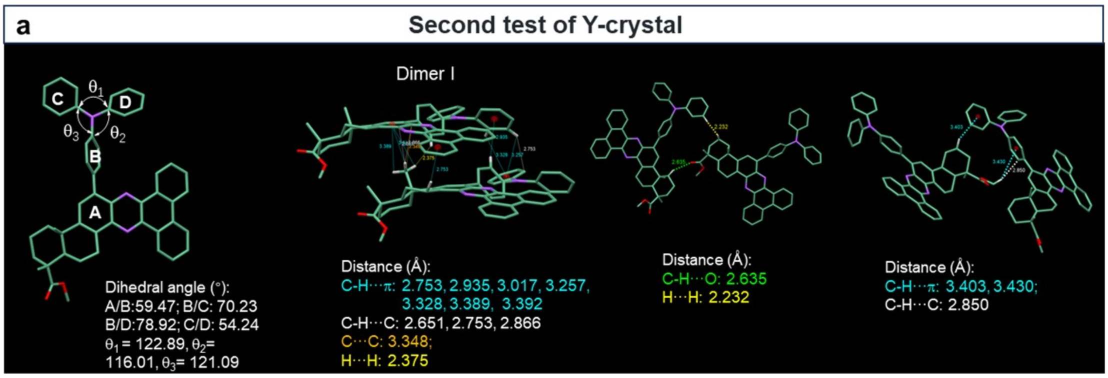

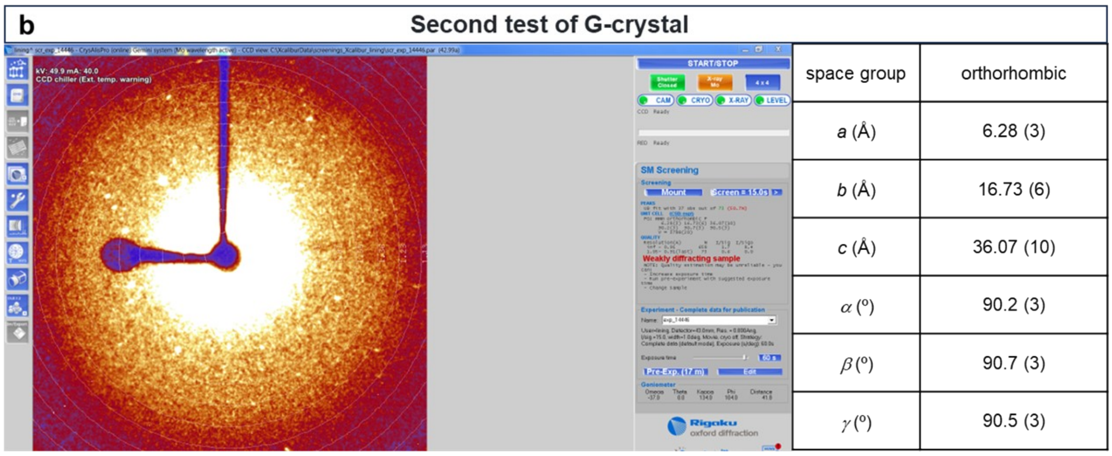

space group	orthorhombic
a (Å)	6.28 (3)
b (Å)	16.73 (6)
c (Å)	36.07 (10)
α (°)	90.2 (3)
β (°)	90.7 (3)
γ (°)	90.5 (3)

Figure S33. (a) The molecular conformations and intermolecular interactions of Y-Crystal of DAQx-BP in the second test. (b) Crystallographic data of G-Crystal of DAQx-BP in the second test.

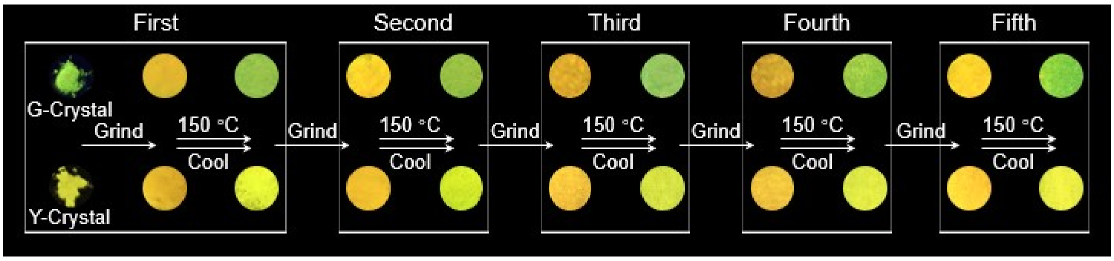

Figure S34. Schematic diagram of the reversible experiment of G and Y crystal photochromism.

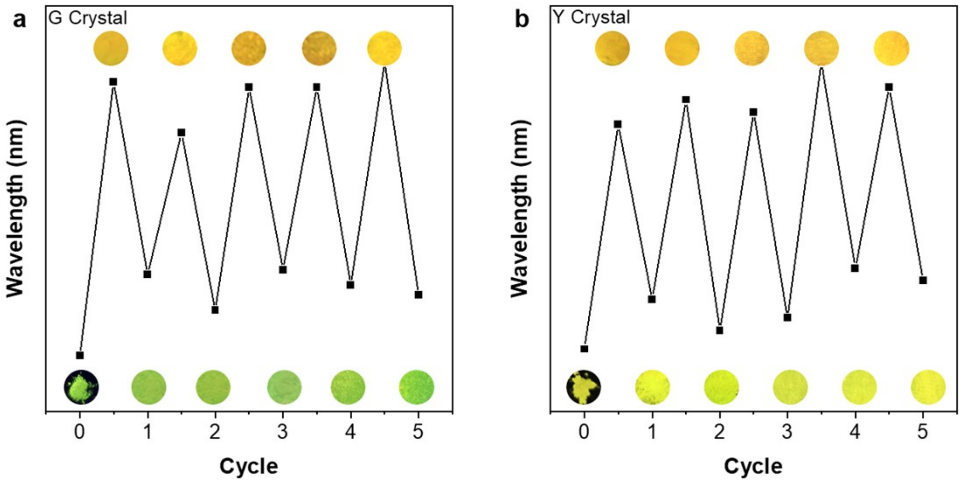

Figure S35. Reversible photochromic experiment PL spectra of (a) G and (b) Y crystals.

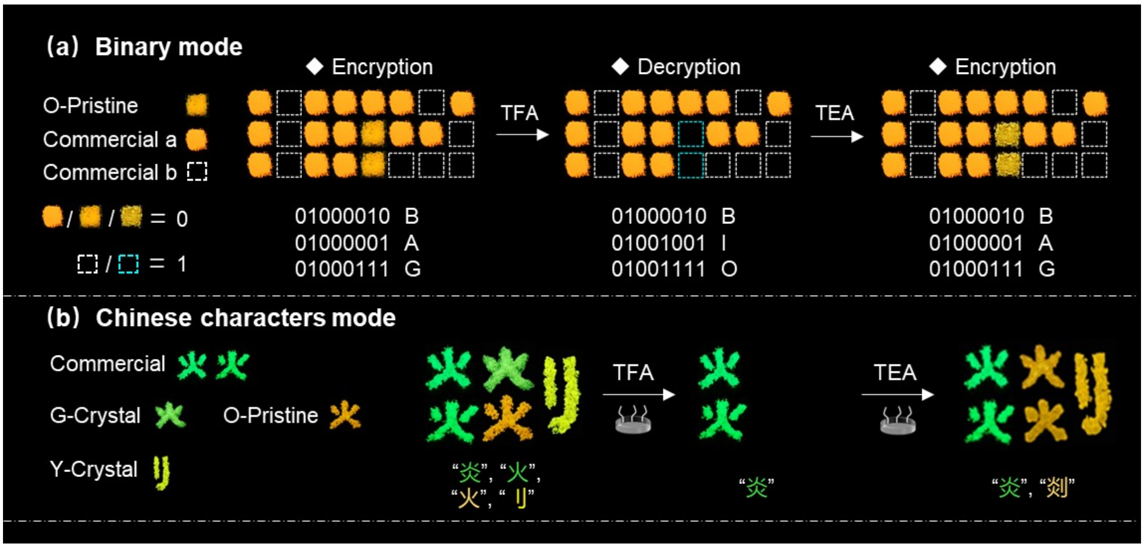

Figure S36. The proof-of-concept demonstrations of encryption-decryption based on DAQx-BP with (a) binary mode, (b) Chinese characters mode.

Reference:

[1] T.-T. Miao, X.-B. Tao, D.-D. Li, H. Chen, X.-Y. Jin, Y. Geng, S.-F. Wang, W. Gu, Synthesis and biological evaluation of 2-aryl-benzimidazole derivatives of dehydroabietic acid as novel tubulin polymerization inhibitors. *RSC Adv.*, 2018, **8**, 17511-17526.
